# Similarities and differences in d^6^ low-spin ruthenium, rhodium and iridium half-sandwich complexes: synthesis, structure, cytotoxicity and interaction with biological targets

**DOI:** 10.1007/s00775-019-01665-2

**Published:** 2019-05-21

**Authors:** Agnieszka Gilewska, Barbara Barszcz, Joanna Masternak, Katarzyna Kazimierczuk, Jerzy Sitkowski, Joanna Wietrzyk, Eliza Turlej

**Affiliations:** 1 Institute of Chemistry, Jan Kochanowski University, 15G Świętokrzyska Str., 25-406 Kielce, Poland; 2 Department of Inorganic Chemistry, Faculty of Chemistry, Gdańsk University of Technology, 11/12 G. Narutowicza Str., 80-233 Gdańsk, Poland; 3 Institute of Organic Chemistry, Polish Academic of Science, Kasprzaka 44/52, 01-224 Warsaw, Poland; 4 National Medicines Institute, Chełmska 30/34, 00-725 Warsaw, Poland; 5 Institute of Immunology and Experimental Therapy, Polish Academy of Sciences, 12 Weigla Str., 53-114 Wrocław, Poland

**Keywords:** Organoruthenium(II), Organorhodium(III), Organoiridium(III), 2,2′-Biimidazole, Antitumor activity

## Abstract

In this paper, we discussed the similarities and differences in d^6^ low-spin half-sandwich ruthenium, rhodium and iridium complexes containing 2,2′-biimidazole (H_2_biim). Three new complexes, {[RuCl(H_2_biim)(η^6^-*p*-cymene)]PF_6_}_2_·H_2_O (**1**), [(η^5^-Cp)RhCl(H_2_biim)]PF_6_ (**2**), and [(η^5^-Cp)IrCl(H_2_biim)]PF_6_ (**3**), were fully characterized by CHN, X-ray diffraction analysis, UV–Vis, FTIR, and ^1^H, ^13^C and ^15^N NMR spectroscopies. The complexes exhibit a typical pseudooctahedral piano-stool geometry, in which the aromatic arene ring (*p*-cymene or Cp) forms the seat, while the bidentate 2,2′-biimidazole and chloride ion form the three legs of the piano stool. Moreover, the cytotoxic activities of the compounds were examined in the LoVo, HL-60, MV-4-11, MCF-7 human cancer cell lines and BALB/3T3 normal mouse fibroblasts. Notably, the investigated complexes showed no cytotoxic effects towards the normal BALB/3T3 cell line compared to cisplatin, which has an IC_50_ value of 2.20 µg. Importantly, **1** displayed the highest activity against HL-60 (IC_50_ 4.35 µg). To predict a binding mode, we explored the potential interactions of the metal complexes with CT-DNA and protein using UV absorption and circular dichroism. The obtained data suggest that the complexes could interact with CT-DNA via an outside binding mode. Moreover, binding of the complexes with the GSH via UV–Vis and ESI mass spectra was determined. Comparative studies have shown that the rhodium complex (**2**) is the most GSH reactive, which is probably responsible for its deactivation towards LoVo and MCF-7 tumour cells. The influence of the metal ion on the biological activity of isostructural Rh(III) and Ir(III) complexes was an important goal of the presented investigation.

**Electronic supplementary material:**

The online version of this article (10.1007/s00775-019-01665-2) contains supplementary material, which is available to authorized users.

## Introduction

By screening the literature connected to the effort of scientists attempting to resolve the problems associated with cancer treatment, it should be noted that organometallic, bioinorganic and bioorganic chemistry are important branches of this subject. Contemporary medicine, including chemotherapy, is mainly based on Pt-anticancer drugs to overcome the following important problems: (1) drug resistance, (2) toxic side effects, and (3) lack of activity against several types of cancer [1]. Therefore, scientists have been paying special attention to organometallic complexes in recent years when looking for new chemotherapeutics. Special efforts have been focused on ruthenium complexes, such as [ImH][*trans*-Ru(Im)_2_Cl_4_] (KP1019, Im = imidazole) [2, 3] and [ImH][*trans*-Ru(DMSO)(Im)Cl_4_] (NAMI-A, Im = imidazole, DMSO = dimethylsulfoxide) [4, 5], as anti-tumour and antimetastatic agents, respectively. This research has inspired the search for metallodrugs based on an increasing range of transition metals, such as gold, rhodium, iridium, and osmium. Going in this direction, the group of Sadler and Dyson [6, 7] developed ruthenium half-sandwich complexes that are characterized by similar ligand exchange kinetics to platinum(II) anticancer drug candidates and whose biological activities are mainly affected by the nature of the chelating ligands (two legs of the piano-stool) and arene ligands that form the piano-stool complexes. Alessio et al. [8] worked on a categorization of metal anticancer compounds based on their modes of action and classified these complexes into groups of functional compounds. Additionally, the anticancer activities of functional compounds will depend on their metal centres, electron configuration (depending on the place in which the metal is situated in the periodic table), hard-soft nature and oxidation state. Interestingly, a study from Sadler’s group [9–11] focused on the biological activity of isostructural piano-stool complexes that only differed in the metal centre showed that the ruthenium(II) compound RM175 and its osmium(II) analogue AFAP51 exhibited significant differences in their anticancer activities in vitro and in vivo. In contrast, Dyson et al. [12] compared the osmium analogue of RAPTA-C, as well as the CpRh(III) and CpIr(III) derivatives to the NAMI-A Ru(III) complex, and demonstrated that both the Rh(III) and Ir(III) compounds are inactive. Moreover, Samuelson et al. [13] demonstrated that the hydrogen bond stability of half-sandwich Ru(II) complexes with heterocycles plays an important role in the anticancer mechanism. The presence of strong hydrogen bonding was better at stabilizing the complexes. However, in the absence of stabilizing interactions, the ligand dissociates in solution, which was confirmed by NMR spectroscopy studies. These structure–activity relationships based on hydrogen bonding may have modulated the drug action mechanism at both the cellular and molecular levels [14, 15]. During past several years, we have been working towards Ru(II) piano-stool complexes, including {[RuCl(L^1^)(η^6^-*p*-cymene)]PF_6_}_2_·H_2_O (L^1^ = 2,2′-bis(4,5-dimethylimidazole)) [16], [(η^6^-*p*-cymene)RuCl(2,2′-PyBIm)]PF_6_ [17] and Rh(II) dimeric complex (Et_3_NH)_2_[Rh_2_(µ_2_-L)_4_Cl_2_] (L^−^ = thiophene-2-carboxylate) [18], as well as focusing on their cytotoxic activity and DNA/protein binding. The main goal of our efforts was to obtain a potential chemotherapeutic that does not affect normal cell lines but possesses better cytotoxic activity against cancer cell lines. In continuation of our work, which was inspired by Sadler and Dyson [9–12], we focused on determining the influence of complexes that differ only in their central metal ion [Rh(III) and Ir(III)] on cytotoxic activity. Additionally, we attempted to estimate whether the presence of hydrogen bonds and noncovalent interactions (HS analysis) influenced the stability and cytotoxic activity of the obtained complexes. Therefore, the main purposes of the present work were as follows: (1) synthesis and full physicochemical characterization of d^6^ low-spin arene ruthenium and isostructural pentamethylcyclopentadienyl rhodium and iridium half-sandwich complexes with 2,2′-biimidazole, (2) determination and comparison of lipophilicity and in vitro cytotoxic properties, and (3) investigation of the types of interactions with biological targets (DNA, HSA, and GSH). To achieve these goals, elemental analysis; single-crystal X-ray diffraction; ^1^H, ^13^C, and ^15^N NMR; and infrared and UV–Vis spectroscopy methods were successfully applied. Moreover, the potential cytotoxic effects of the {[RuCl(H_2_biim)(η^6^-*p*-cymene)]PF_6_}_2_·H_2_O (**1**), [(η^5^-Cp)RhCl(H_2_biim)]PF_6_ (**2**), and [(η^5^-Cp)IrCl(H_2_biim)]PF_6_ (**3**) complexes were examined towards the LoVo (colorectal adenoma), MV-4-11 (myelomonocytic leukaemia), MCF-7 (breast adenocarcinoma), HL-60 (promyelocytic leukaemia) and normal BALB/3T3 (mouse fibroblast) cell lines. With the aim of gaining deeper insights into the mechanisms of action, we investigated the interactions with DNA, HSA via UV–Vis and CD methods. Moreover, binding of the complexes with the GSH via UV–Vis and ESI mass spectra was determined.

## Experimental section

### Materials and physical measurements

[(η^6^-*p*-Cymene)Ru(µ-Cl)Cl]_2_ (97%), [CpRh(μ-Cl)Cl]_2_ (99%), [CpIr(μ-Cl)Cl]_2_ (99%), 2,2′-biimidazole (H_2_biim), NH_4_PF_6_ (99%), cisplatin, 5,5′-dithio-bis(2-nitrobenzoic acid) (DTNB) (99%), human serum albumin (HSA) (lyophilized powder, < 0.007% fatty acid), l-glutathione reduced form (GSH) (lyophilized powder, > 98%) were purchased from Sigma-Aldrich. Analytical grade solvents were purchased from Chempur. Tris(hydroxymethyl)aminomethane-HCl (5 mM Tris–HCl/50 mM NaCl, pH 7.2) and PBS (10 mM, pH = 7.4) buffer were prepared using MiliQ water.

Elemental analysis was run on an Elementar Vario Micro Cube analyzer. The FTIR spectra were recorded on a Nicolet 380 spectrophotometer in the spectral range 4000–500 cm^−1^ using the ATR-diffusive reflection method. UV–Vis measurements in methanolic solutions were performed on UV–Vis spectrophotometer (V-630 Jasco) using 1 cm cuvettes against methanol as reference solutions. All absorbance measurements were recorded at room temperature and the concentrations 1.0 × 10^−4^ for ligand (H_2_biim) and complex **1**, **2**, **3**. Circular dichroism spectra were recorded with a Jasco J-815 spectropolarimeter (Jasco Inc.) equipped with a Jasco Peltier-type temperature controller. The ^1^H, ^13^C and ^15^N NMR spectra were recorded at 25 °C in DMSO-d_6_ solutions on a Varian VNMRS-600 (^1^H 600 MHz, ^13^C 150 MHz, ^15^N 43.5 MHz) spectrometer equipped with a 5-mm PFG AutoXID (^1^H/X^15^N-^31^P) probe. Standard pulse sequences were used except the ^1^H-{^15^N} correlation. Gradient-enhanced IMPACT-HMBC [19] ^1^H-{^15^N} correlation spectra were optimized for a coupling constant of 6 Hz with the following experimental conditions: an acquisition time of 0.3 s, spectral windows of 8000 (F2) and 18,000 (F1) Hz, 2700 complex data points in t2, 256 or 512 complex data points in t1, 4 scans per increment, 30 ms WURST-2 mixing sequence centered within the 60 ms preparation interval (ASAP2) and a 150 Ernst angle as the excitation pulse [20]. The data were processed with linear prediction in t1 followed by zero filling in both dimensions. Gaussian weighting functions were applied in both domains prior to Fourier transformation. All the spectra were referenced according to IUPAC recommendations [21]. The ESI mass spectra of free GSH, GSH-cisplatin, GSH-complexes (**1**–**3**) were recorded on a micrOTOF-Q II (Brucker) equipped with syringe pump. The dry gas flow rate was at 4.0 l/min; the dry heater was operated at 180 °C; the capillary voltage was set at 4.500 V and collision energy was variable and set between 2 and 10 eV. The sample solutions were prepared in acetonitrile–methanol mixture (1:1).

### Crystal structure determination and refinement

Single-crystal X-ray diffraction data of compounds **1–3** were collected at 120(2) K on a Stoe IPDS-2T diffractometer with graphite-monochromated Mo-Kα radiation. Data collection and image processing were performed with X-Area 1.75 (STOE & Cie GmbH, 2015) [22]. Intensity data were scaled with LANA (part of X-Area) to minimize differences of intensities of symmetry-equivalent reflections (multi-scan method). The crystal was thermostated in nitrogen stream at 120 K using CryoStream-800 device (Oxford CryoSystem, UK) during the entire experiment. The structure was solved with direct methods and was refined with the SHELX-2016/6 program package [23, 24] with the full-matrix least squares procedure based on F2. The Olex [25] and WingX [26] program suites were used to prepare the final version of CIF files. Diamond [27] was used to prepare the figures. All C–H type hydrogen atoms were attached at their geometrically expected positions and refined as riding on heavier atoms with the usual constraints. In the all compounds, residual electron density is somewhat high and localizes near the heavier Ru/Ir atoms, and is not attributable to an additional atom. A summary of crystallographic data is shown in Table S1. Crystallographic data for structure of **1**, **2** and **3** reported in this paper have been deposited with the Cambridge Crystallographic Data Center as supplementary publications No. CCDC 1881107–1881109. Copies of the data can be obtained free of charge on application to CCDC, 12 Union Road, Cambridge CB2 1EZ, UK (Fax: (+44) 1223-336-033; Email: deposit@ccdc.cam.ac.uk).

### Synthesis of complexes **1**–**3**

#### Preparation of {[RuCl(H_2_biim)(η^6^-*p*-cymene)]PF_6_}_2_·H_2_O (**1**)

A solution of the precursor compound [(η^6^-*p*-cymene)Ru(µ-Cl)Cl]_2_ (0.25 mmol, 0.153 g), ligand 2,2′-biimidazole (0.5 mmol, 0.067 g) and NH_4_PF_6_ (0.5 mmol, 0.0815 g) was dissolved in a 20 ml mixed solution (CH_3_OH:CH_2_Cl_2_; 3 V:1 V) with one drop of H_2_O. The reaction mixture was refluxed for 12 h (~ 65 °C). After 2 weeks, the resulting orange crystals suitable for single-crystal X-ray diffraction were filtered and dried in a vacuum box. The crystals were collected at 75% yield. Anal. Calc. (%) for C_32_H_42_Cl_2_F_12_N_8_OP_2_Ru_2_: C, 33.95; H, 3.67; N, 10.19. Found: C, 33.42; H, 3.50; N, 10.22; ^1^H NMR (600 MHz, DMSO-d_6_, *δ* ppm): 1.00 [6H, d, (C_28/29_**H**_3_)_2_C_27_H)]; 2.61 [1H, m, (C_28/29_H_3_)_2_C_27_**H**)]; 6.00 [2H, C_23_**H**, C_25_**H**, (*p*-cymene)]; 5.80 [2H, C_22_**H**, C_26_**H**, (*p*-cymene)]; 2.07 (3H, s, C_30_**H**_3_); 7.84 (2H, s, C_4/4′_**H**); 7.50 (2H, s, C_5/5′_**H**); 12.91 (2H, s, N_1/1′_**H**). ^13^C NMR (150 MHz, DMSO-d_6_, *δ* ppm): 22.1 (**C**_28_, **C**_29_); 30.9 (C_27_); 102.6 (C_24_); 83.3 (**C**_23_, **C**_25_); 81.7 (**C**_22_, **C**_26_); 99.8 (**C**_21_); 18.7 (**C**_30_); 138.2 [**C**_2/2′_(H_2_biim)]; 131.8 [**C**_4/4′_(H_2_biim)]; 121.1 [**C**_5/5′_(H_2_biim)]. ^15^N NMR (43.5 MHz, DMSO-d_6_, *δ* ppm): − 218.5 (**N**_1/1′_H); − 181.7 (**N**_3/3′_). IR (cm^−1^): 3260(br), 3145(w), 1527(ms), 1468(ms), 1321(ms), 1188(ms), 1093(ms), 849(vs), 754(ms), 679(ms), 557(s).

#### Preparation of [(η^5^-Cp)RhCl(H_2_biim)]PF_6_ (**2**)

A mixture of the precursor compound [CpRh(μ-Cl)Cl]_2_ (0.1 mmol, 0.0618 g) in 5 ml of methanol was added dropwise to a 15 ml mixed solution of the ligand 2,2′-biimidazole (CH_3_OH:CH_2_Cl_2_; 3 V:1 V) (0.2 mmol, 0.026 g) and NH_4_PF_6_ (0.25 mmol, 0.0407 g). The reaction mixture was stirred and refluxed for 10 h. After that step, the solution was allowed to stand at room temperature to crystallize. The resulting complex was filtered off and dried in a vacuum box after a week. The crystals were collected at 62% yield. Anal. Calc. (%) for C_16_H_21_ClF_6_N_4_PRh: C, 34.77; H, 3.83; N, 10.13. Found: C, 34.20; H, 3.51; N, 9.64; ^1^H NMR (600 MHz, DMSO-d_6_, *δ* ppm): 1.68 [15H, (Cp–C_11–15_**H**_3_)]; 7.56 (2H, s, C_4/4′_**H**); 7.54 (2H, s, C_5/5′_**H**); 12.90 (2H, s, N_1/1′_**H**). ^13^C NMR (150 MHz, DMSO-d_6_, *δ* ppm): 9.3 (Cp–**C**_11–15_H_3_); 95.4 (Cp–**C**_6–10_) (*J* = 8.2 Hz); 138.2 [**C**_2/2′_(H_2_biim)]; 128.2 [**C**_4/4′_(H_2_biim)]; 121.4 [**C**_5/5′_(H_2_biim)]. ^15^N NMR (43.5 MHz, DMSO-d_6_, *δ* ppm): − 218.7 (**N**_1/1′_H); − 173.8 (**N**_3/3′_) (*J* = 18.6 Hz). IR (cm^−1^): 3169(w), 1531(ms), 1433(ms), 1179(ms), 1095(ms), 1021(ms), 836(vs), 746(ms), 608(ms), 557(s).

#### Preparation of [(η^5^-Cp)IrCl(H_2_biim)]PF_6_ (**3**)

Complex **3** was prepared similar to complex (**2**) using [CpIr(μ-Cl)Cl]_2_ (0.1 mmol, 0.0797 g) as the metal source. The yellow crystals appeared after a week and were collected at 49% yield. Anal. Calc. (%) for C_16_H_21_ClF_6_N_4_PIr: C, 29.93; H, 3.29; N, 8.73. Found: C, 30.17; H, 3.03; N, 8.78; ^1^H NMR (600 MHz, DMSO-d_6_, *δ* ppm): 1.69 [15H, (Cp–C_11–15_**H**_3_)]; 7.56 (2H, s, C_4/4′_**H**); 7.54 (2H, s, C_5/5′_**H**); 13.15 (2H, s, N_1/1′_**H**). ^13^C NMR (150 MHz, DMSO-d_6_, *δ* ppm): 9.1 (Cp–**C**_11–15_H_3_); 87.1 (Cp–**C**_6–10_); 139.9 [**C**_2/2′_(H_2_biim)]; 127.8 [**C**_4/4′_(H_2_biim)]; 121.8 [**C**_5/5′_(H_2_biim)]. ^15^N NMR (43.5 MHz, DMSO-d_6_, *δ* ppm): − 218.1 (**N**_1/1′_H); − 189.7 (**N**_3/3′_). IR (cm^−1^): 3172(w), 1533(ms), 1458(ms), 1181(ms), 1093(ms), 1025(ms), 835(vs), 740(ms), 652(ms), 565(s).

### Cells and cytotoxicity assay

The cytotoxicity of the investigated complexes (**1–3)** was determined by microculture with sulforhodamine B SRB (all adherent cells) and MTT ([3-(4,5-dimethyl(thiazol-2-yl)-2,5-diphenyltetrazoliumbromide)]) [for cells in suspension (leukaemia cells)] assays [28, 29] with the following four tumour cell lines: colorectal adenoma (LoVo), promyelocytic leukaemia (HL-60), myelomonocytic leukaemia (MV-4-11), and breast adenocarcinoma (MCF-7), as well as a healthy mouse fibroblast (BALB/3T3) cell line. All tested cell lines were obtained from the American Type Culture Collection (Rockville, MD, USA) and maintained at the Cell Culture Collection of the Institute of Immunology and Experimental Therapy (Wrocław, Poland). The colorectal adenoma cells were cultured in a mixture of F12K-ATCC medium. The breast cancer cells were bred in Eagle and MEM medium supplemented with glutamine and insulin (Sigma-Aldrich). The myelomonocytic leukaemia carcinoma cells were grown in a mixture of RPMI 1640 w/GLUTAMAX-I medium (Gibco) supplemented with pyruvate (Sigma-Aldrich). The promyelocytic leukaemia carcinoma cells were grown in a mixture of RPMI 1640 w/GLUTAMAX-I medium (Gibco) supplemented with glucose (Sigma-Aldrich). The fibroblast cells were cultured in DMEM medium (Gibco) supplemented with glutamine (Sigma-Aldrich). Moreover, all the tested media were supplemented with 10% HealthCare foetal bovine serum (Sigma-Aldrich), streptomycin (Sigma-Aldrich) and penicillin (Polfa Tarchomin). All cell lines were grown at 37 °C in a humidified atmosphere with 5% CO_2_. The cells were plated in 96-well plates (Sarstedt, Germany) at a density of 10^4^ cells per well and were cultured in 100 µl of culture medium. After a 24 h incubation period, the cells were treated with compound for an additional 72 h. Cisplatin, a platinum-based chemotherapy drug, was used as a standard comparison treatment. The ruthenium complex stock solution was always freshly prepared by dissolving 1 mg of compound in 100 µl DMSO. After that step, the obtained solution was diluted in culture medium to reach the required concentrations (ranging from 0.1 to 100 µg/ml). The final concentration of DMSO in the cell culture medium did not exceed 0.1% (v/v), which was shown to have no effect on cell growth. The in vitro cytotoxic activity results were expressed as IC_50_ values, which is the concentration of compound (in µg) that inhibits the tumour cell proliferation rate by 50% compared with the untreated control cells. The results were calculated from 2 to 3 independent experiments.

### Determination of stability

The stability of complexes **1**, **2** and **3** was examined in a mixture of one drop of DMSO and 5 mM Tris–HCl/50 mM NaCl buffer at pH 7.2 and determined by UV–Vis spectroscopy. The solution was maintained at room temperature (rt). After 0 and 24 h, electronic spectra were recorded with a Jasco 630 UV–Vis spectrophotometer. To confirm the stability of the complexes, ^13^C NMR spectra were recorded in DMSO at room temperature at two different time points: immediately after dissolution and after 24 h.

### Partition coefficient

The lipophilicity of **1**, **2**, **3** was determined using the shake-flask method [30]. The compounds (ca. 10^−4^ M) were dissolved in aqueous phase (5 mM Tris–HCl/50 mM NaCl buffer pH 7.2), and this solution was added to *n*-octan-1-ol. The mixtures were mechanically mixed in tubes for 1 h, 12 h, 24 h, 30 h and 48 h until reaching solubility equilibrium followed by centrifugation (5000 rpm, 15 min). After separation, the phases were analysed by UV–Vis spectroscopy to determine the amount of the compound in the water phase. The absorption values before and after shaking were compared. The phase water–octanol equilibrium was established after 30 h for complexes **2** and **3** and after 24 h for complex **1**. The partition coefficient (log *P*) for each compound was calculated based on the Lambert–Beer Law to determine the log *P* values. The procedures were repeated six times for each complex, and the values of the log *P* are the mean ± standard deviation.

### Interaction with biomolecules

#### Calf thymus DNA

Binding of {[RuCl(H_2_biim)(η^6^-*p*-cymene)]PF_6_}_2_·H_2_O (**1**), [(η^5^-Cp)RhCl(H_2_biim)]PF_6_ (**2**) and [(η^5^-Cp)IrCl(H_2_biim)]PF_6_ (**3**) with polymeric CT-DNA was evaluated via UV–Vis absorption titrations and CD spectroscopy. The stock solutions of the complexes and CT-DNA were prepared using 5 mM Tris–HCl/50 mM NaCl buffer at pH 7.2. The DNA concentration per nucleotide was determined by UV absorption measurements using the molar absorption coefficient *ε*_258_ = 6 600 M^−1^ cm^−1^ [31]. Stock solutions were stored at 4 °C and used within 7 days. All experiments were performed by maintaining the concentration of complexes constant (300 µM) while varying the CT-DNA concentration from 0 to 300 µM. The CD spectra of the solutions were measured after 24 h and 48 h of incubation at 37 °C in the range from 200 to 350 nm under a dry nitrogen atmosphere. In contrast, the UV–Vis spectra were recorded after 40 min and 24 h of incubation at room temperature in the range from 200 to 600 nm. From the absorption titration data, the intrinsic binding constant (*K*_b_) of the metal complexes with CT-DNA was determined using the equation given as:

$$\left[ {\text{DNA}} \right]/\left( {\varepsilon_{\text{a}} - \varepsilon_{\text{f}} } \right)\, = \,\left[ {\text{DNA}} \right]/\left( {\varepsilon_{\text{b}} - \varepsilon_{\text{f}} } \right)\, + \, 1/K_{\text{b}} \left( {\varepsilon_{\text{b}} - \varepsilon_{\text{f}} } \right),$$

where [DNA] is the concentration of DNA in base pairs, *ε*_a_ is the extinction coefficient of the complex at a given DNA concentration, *ε*_f_ is the extinction coefficient of the complex in free solution, and *ε*_b_ is the extinction coefficient of the complex when fully bound to DNA. A plot of [DNA]/(*ε*_a_ − *ε*_f_) versus [DNA] gave a slope and an intercept equal to 1/(*ε*_b_ − *ε*_f_) and 1/*K*_b_/(*ε*_b_ − *ε*_f_), respectively. The intrinsic binding constant *K*_b_ is the ratio of the slope to the intercept.

#### Human serum albumin

The interaction of {[RuCl(H_2_biim)(η^6^-*p*-cymene)]PF_6_}_2_·H_2_O (**1**), [(η^5^-Cp)RhCl (H_2_biim)]PF_6_ (**2**) and [(η^5^-Cp)IrCl(H_2_biim)]PF_6_ (**3**) with HSA was studied by UV–Vis absorption titrations at 210–320 nm wavelengths; *λ*_max_ was recorded at 278 nm (1 cm cuvette). The CD spectroscopy was performed from 220 to 300 nm (0.2 cm cuvette) in 5 mM Tris–HCl/50 mM NaCl buffer at pH 7.2. Solid HSA was dissolved in Tris–HCl/NaCl buffer (5/50 mM at pH 7.2) and the resulting stock HSA solution was kept at 4 °C for a maximum of 1 day. All the experiments involved 16.5 µM HSA (UV–Vis spectroscopy) or 10 µM HSA (CD spectroscopy) and increasing amounts of the complexes dissolved in Tris–HCl/NaCl buffer (pH = 7.2). The CD spectra of these solutions were measured after 24 h of incubation at 37 °C, while the UV–Vis spectra were measured after 40 min and 24 h of incubation at room temperature. The concentrations of the stock solution of complexes **1**, **2**, and **3** were 1.5 × 10^−4^ M (UV–Vis spectroscopy) and 3 × 10^−4^ M (CD spectroscopy). The HSA concentration was determined spectrophotometrically using an extinction coefficient *ε*_280_ = 36 600 M^−1^ cm^−1^ [32].The plot of (*ε*_a_ − *ε*_f_) (where *ε*_f_ is the initial absorbance of free HSA at 278 nm and *ε*_a_ is the absorbance of HSA in the presence of different concentrations of the complex) versus [complex] is a linear curve, and the binding constant (*K*_b_) can be obtained from the ratio of the intercept to the slope. The results of HSA interaction with complexes via CD spectroscopy were expressed as mean residue ellipticity (MRE) in deg cm^2^ dmol^−1^. The value of MRE can be obtained using the equation MRE = [*Θ*]/(*nlC* × 10), where [*Θ*] is the CD in millidegrees obtained from the spectra (at 208 nm), *n* is the number of amino acid residues (585 for HSA), *l* is the path length of the cell (0.2 cm), and *C* is the mol fraction of the protein. Then, the helical content of HSA can be calculated from the [*Θ*] value at 208 nm according to the equation  % helix = {(− *MRE*_208_ − 4000)/(33000 − 4000)} × 100 as described by Liu et al. [33].

#### Glutathione

The reactivity towards glutathione was determined using Ellman’s method [34–36] to measure the unreacted GSH concentration after incubation with the complexes. The procedure is based on the reaction of the thiol with DTNB to give the mixed disulfide and 2-nitro-5-thiobenzoic acid which was quantitatively determined by the UV–Vis spectroscopy at 412 nm. Mixtures of 1.0 ml of GSH (sample concentration 0.5–3.0 mM) 1 ml of **1**–**3** complexes (0.5 mM) and 8 ml of H_2_O were incubated at 25 °C in the dark for 30 h. Next 2.5 ml of the reaction mixture was treated with 2.5 ml of DTNB in a phosphate buffer (PBS, 10 mM, pH 7.4) and the absorbance was determined. A comparison with the reference GSH concentration/absorbance relationship allowed the determination of unreacted thiol concentration. The obtained concentrations are the average of three independent measurements. Moreover, to examined products of the reaction GSH with the compounds the ESI mass spectra of free GSH, GSH cisplatin, GSH complexes (**1**–**3**) were recorded.

## Results and discussion

**Fig. 1:**
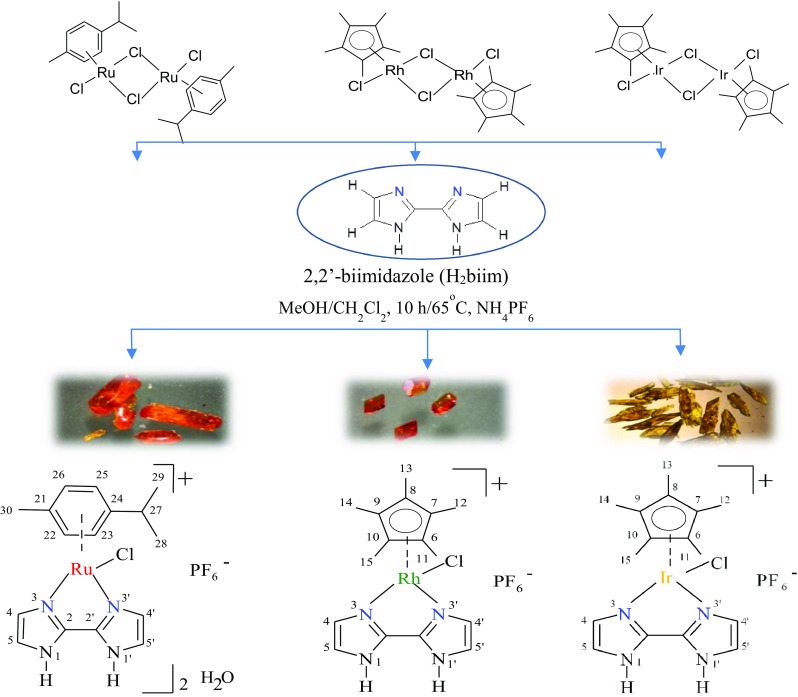
Schematic presentation of the syntheses of complexes **1–3**

The reaction of appropriate metal precursors: dichloro(*p*-cymene)ruthenium(II) dimer [(η^6^-*p*-cymene)Ru(µ-Cl)Cl]_2_, pentamethylcyclopentadienylrhodium(III) dimer [CpRh(μ-Cl)Cl]_2_, pentamethylcyclopentadienyliridium(III) dimer [CpIr(μ-Cl)Cl]_2_, with 2,2-biimidazole ligand (H_2_biim) with appropriate ratio in dry mixture of methanol/dichloromethane resulted in the formation of d^6^ low-spin half-sandwich ruthenium, rhodium and iridium complexes: {[RuCl(H_2_biim)(η^6^-*p*-cymene)]PF_6_}_2_·H_2_O (**1**), [(η^5^-Cp)RhCl(H_2_biim)]PF_6_ (**2**) and [(η^5^-Cp)IrCl(H_2_biim)]PF_6_ (**3**) (Fig. 1). All the complexes were isolated as orange to yellow solids and were found to be stable in air and non-hydroscopic. These complexes are soluble in hot water, methanol, DMSO but insoluble in hexane and diethyl ether.

### Description of the structures

**Fig. 2:**
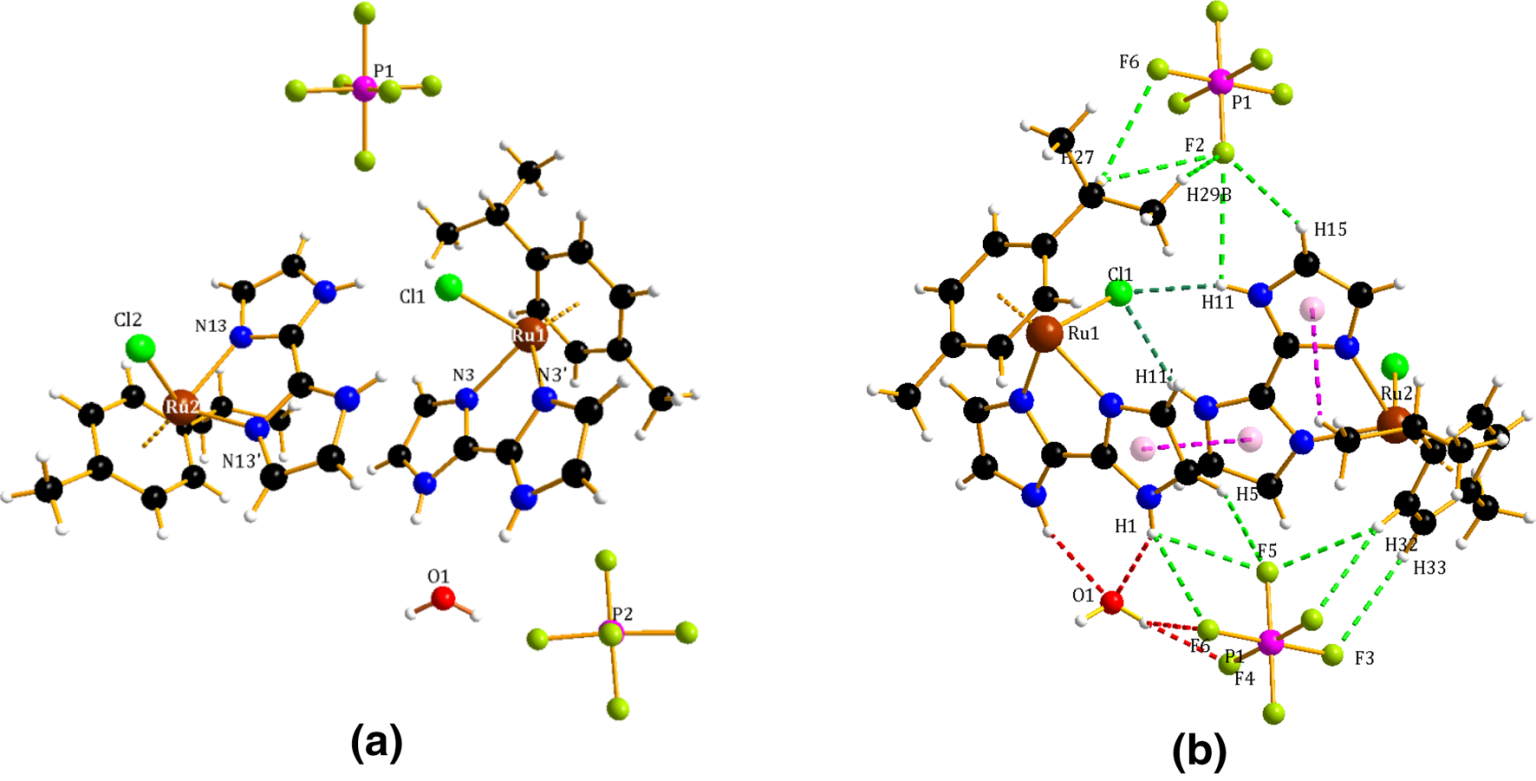
Molecular structure of asymmetric unit of complex **1** (**a**) with marked N–H···O, N/C/O–H···F, N–H···Cl, C–H···*π* and *π*···*π* interaction (**b**)

The crystal structure determination data are summarized in Table S1. The selected bond distances and bond angles are tabulated in Table 1. The perspective views of the crystal structures of the presented complexes are depicted in Fig. 2a. The asymmetric units of **1** contain two cationic complex molecules, two PF_6_^¯^ counterions and one molecule of water. The asymmetric units of complexes **2** and **3** contain a cationic complex molecule and a PF_6_^¯^ counterion. The coordination sphere of each Ru(1) and Ru(2) is built up by an η^6^-*p*-cymene, a chloride and an N^∩^N-κ^2^*N*,*N*′ 2,2′-biimidazole ligand. The distance from ruthenium(II) to the centroid of the *p*-cymene ring is similar to that observed in the literature [37]. The Ru–Cl (2.433(2) and 2.424(3) Å) and Ru–N bond lengths (from 2.093 to 2.115(8) Å) are in the expected range. The structure can be considered as a distorted octahedron because the angles at the ruthenium(II) ions are close to 90° [76.7(3)–86.9(2)°]. The distance observed between the two ruthenium centres in the dimer [7.9687(11) Å] excludes any possible metal–metal interactions.

**Table 1:** Bond lengths and angles for complexes **1**–**3**

1		**2**		**3**	
Distances (Å)					
Ru(1)···Cg1	1.6692(7)	Rh(1)···Cp	1.7609(2)	Ir(1)···Cp	1.7734(5)
Ru(1)–Cl(1)	2.433(2)	Rh(1)–Cl(1)	2.4419(6)	Ir(1)–Cl(1)	2.4408(13)
Ru(1)–N(3)	2.103(8)	Rh(1)–N(1)	2.114(2)	Ir(1)–N(1)	2.109(4)
Ru(1)–N(3′)	2.081(8)	Rh(1)–N(3)	2.117(2)	Ir(1)–N(3)	2.105(4)
Ru(2)···Cg2	1.6680(8)				
Ru(2)–Cl(2)	2.424(3)				
Ru(2)–N(13)	2.115(8)				
Ru(2)–N(13′)	2.093(8)				
Angles (°)					
N(3′)–Ru(1)–N(3)	76.7(3)	N(3)–Rh(1)–N(1)	77.01(9)	N(3)–Ir(1)–N(1)	76.06(14)
N(3′)–Ru(1)–Cl(1)	86.9(2)	N(1)–Rh(1)–Cl(1)	87.42(6)	N(1)–Ir(1)–Cl(1)	85.15(10)
N(3)–Ru(1)–Cl(1)	85.4(2)	N(3)–Rh(1)–Cl(1)	87.04(6)	N(3)–Ir(1)–Cl(1)	85.94(10)
N(13′)–Ru(2)–N(13)	76.2(3)				
N(13′)–Ru(2)–Cl(2)	83.0(2)				
N(13)–Ru(2)–Cl(2)	86.0(2)

The crystal packing of complex **1** shows some intermolecular interactions, including N–H···O, N/C/–H···F, C–H···*π* and *π*···*π*, that contribute to the stability of the compound. Moreover, two symmetrically independent [RuCl(H_2_biim)(η^6^-*p*-cymene)]^+^ cations are held together via *π*···*π* interactions between the parallel-displaced imidazole rings [3.7835(2) Å] and an N–H···Cl interaction [N(11)/(11′)–H(11)/(11′)···Cl(1), 2.37 and 2.45 Å, respectively] (Fig. 2b). Consequently, a chain of hydrogen bonds is created along the crystallographic direction (Fig. S2a), and the crystal structure of the complex exhibits tetrameric units that are separated by water molecules (Fig. S2b).

**Fig. 3:**
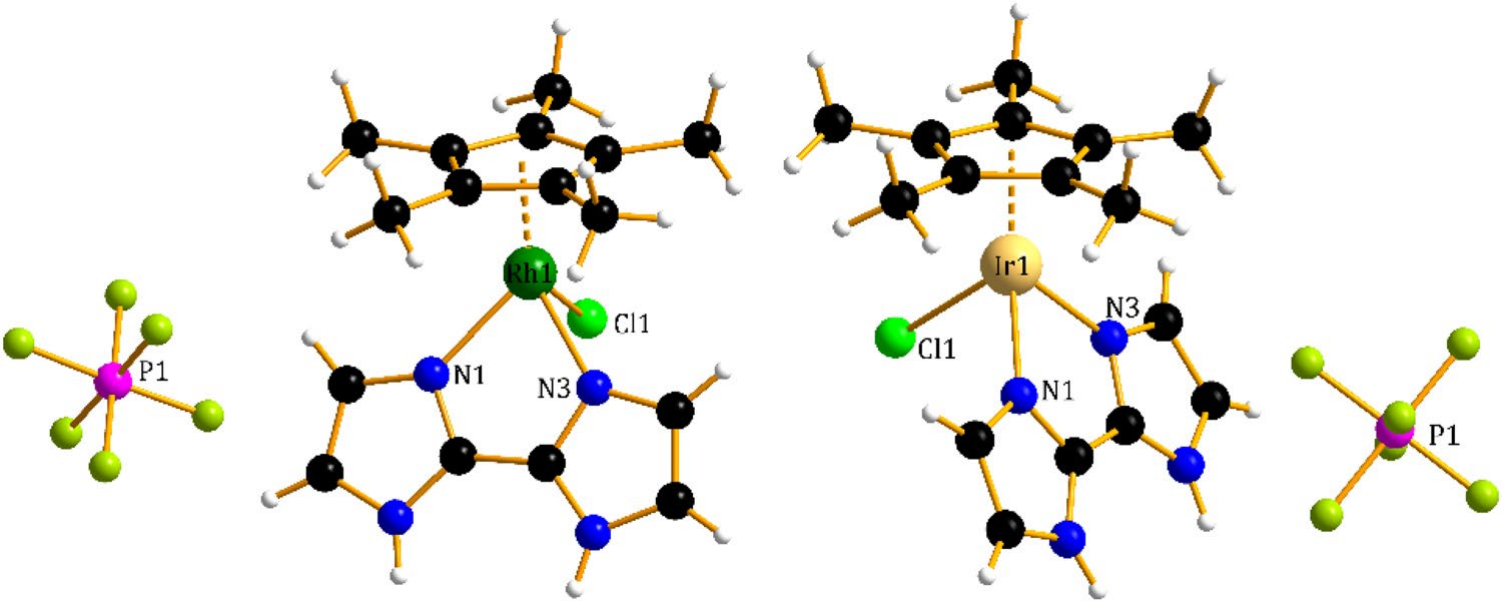
Molecular structures of **2** (left) and **3** (right)

The next two metal complexes with the general formula [(η^5^-Cp)M(H_2_biim)Cl][PF_6_], in which M = Rh(III) (**2**) or Ir(III) (**3**) and H_2_biim represents 2,2′-biimidazole, display the standard “three-leg piano-stool” geometry (Fig. 3). The half-sandwich rhodium(III) complex (**2**) consists of a *π*-bonded η^5^-pentamethylcyclopentadienyl ligand, a doubly σ-bonded 2,2′-biimidazole, as well as a chloride ligand. The Rh–C bond distances in complex **2** [ranging from 2.1311(32) to 2.1632(22) Å] are consistent with previous reports [37, 38]. The structure of the analogous iridium(III) complex (**3**) shows slightly longer distances between the metal ion and the Cp ring [2.1445(47)–2.1794(44) Å]. Accordingly, replacement of the rhodium with iridium in compound **3** surprisingly compresses the M–N bond lengths of the chelating κ^2^*N*,*N′*–H_2_biim ligand (see Table 1) and is similar to those found in other Rh and Ir-η^5^-Cp compounds [37, 38].

Thus, the M(III)···Cg and M(III)-*N*,*N*-H_2_biim bonds are similar in complexes **2** and **3**; however, careful inspection of Table 1 shows that there are some differences. The slightly longer Ir···Cg distance [1.7734(5) Å] compared with the Rh···Cg distance [1.7609(2) Å] and the slightly shorter Ir–N and Rh–N distances suggest stronger interactions between the iridium(III) centre and the N^∩^N-2,2′-biimidazole ligand compared to the stronger interactions between the rhodium(III) ion and the Cp ring. Additionally, the M–Cl bond distance in both complexes **2** and **3** [equal to 2.4419(6) and 2.4408(13) Å, respectively] is relatively longer than that in previously reported complexes [37, 38]. In the crystal packing of the rhodium and iridium complexes, we can observe similar *π*···*π* interactions between the imidazole rings and a relatively short C–H···F or N–H···Cl noncovalent interaction between the ligands and the anions (PF_6_^−^, Cl^−^) (Fig. S3, Table S2).

### Hirshfeld surface analysis

**Fig. 4:**
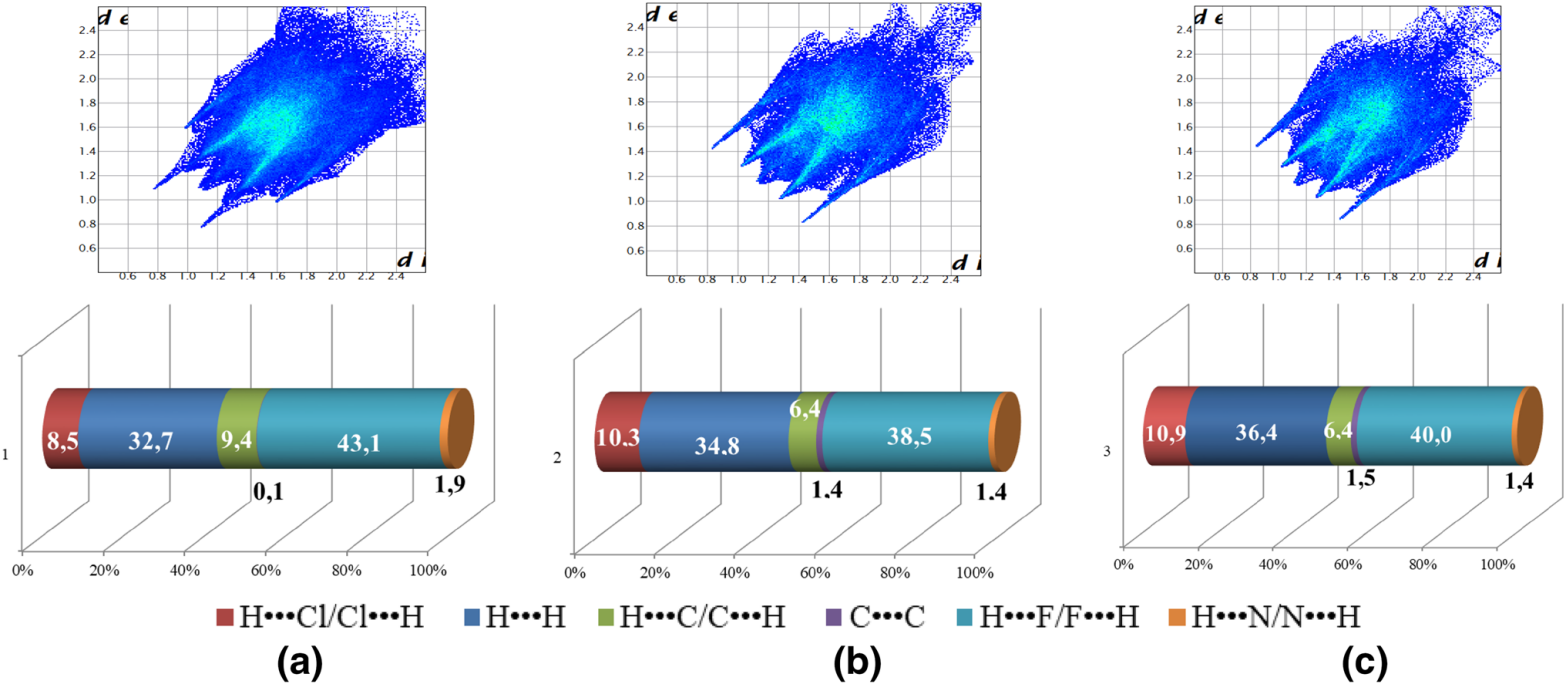
2D fingerprint plots of the most significant intermolecular interactions for **1** (**a**), **2** (**b**) and **3** (**c**) complexes with percentage of interaction

The purpose of examining the interactions in the crystal structures is the opportunity to form noncovalent interactions in biological systems; these interactions not only provide detailed information regarding close contacts but also regarding more distant interactions for **1**, **2**, and **3** through Hirshfeld surface (HS) analysis and the obtained associated 2D fingerprint plots. For the Ru(II) (**1**), Rh(III) (**2**), and Ir(III) (**3**) complexes, the H···F and H···H interactions (C–H···F according to Fig. 4) strongly contribute to the crystal packing. The smaller shares (see Fig. 4 for details) are attributed to the H···Cl, C···H, C···C and N···H interactions, respectively.

The above-mentioned data pointed out that the presence of N–H/C–H hydrogen bond donors in complexes favours multiple noncovalent interactions (especially for **1**), and probably influenced solution stability. It should be underlined that compounds stability in solution is significantly affected by strong bonding chelating ligand (H_2_biim) and the inertness of metal ions [Rh(III), Ir(III)].

#### Spectral studies of the complexes

NMR, UV–Vis and FTIR spectroscopy techniques as well as elemental analysis were used to characterize the complexes. The analytical data from these complexes are consistent with the formulations.

#### NMR spectroscopy

Formation of **1**–**3** has been deduced from ^1^H, ^13^C and ^15^N NMR spectral studies. The resulting spectra are depicted in Figs. S5–S8 in the Electronic Supplementary Materials (ESM); the ensuing data are summarized in the experimental section, and some of the data are presented in Table 2.

**Table 2:** ^1^H, ^13^C and ^15^N NMR chemical shifts of **1**–**3** complexes in DMSO-d_6_

Compound	*δ*H4	*δ*H5	*δ*C4	*δ*C5	*δ*Cp–C/p-cymene-C_(Ru)_	*δ*N3	*δ*N1
**1**	7.84(+ 0.73)	7.50(+ 0.52)	131.8(+ 3.1)	121.1(+ 3.2)	102.6	− 181.7	− 218.5
**2**	7.56(+ 0.45)	7.54(+ 0.56)	128.2(− 0.5)	121.4(+ 3.5)	95.4	− 173.8	− 218.7
**3**	7.56(+ 0.45)	7.54(+ 0.56)	127.8(− 0.5)	121.8(+ 3.5)	87.1	− 189.7	− 218.1
H_2_biim	7.11	6.98	128.7	117.9	–		

The coordination shifts (∆_coord_) are shown in parentheses

Δ_coord._ = *δ*_complex_ − *δ*_ligand_; Cp_(Rh, Ir)_-η^5^-pentamethylcyclopentadienyl

^13^C and ^15^N NMR are interesting to compare the strength of the coordination (by Lewis acids) of the rhodium and iridium central metal ions in the obtained complexes (**2** and **3**, respectively). Both the metal ions are coordinated by the same η^5^-pentamethylcyclopentadienyl ligand (Cp). Considering the *δ*Cp–C, we noticed that the *π*-donating effect is much more effective in the case of the rhodium complex (shift *δ* = 95.4 ppm) compared to the iridium complex (shift *δ* = 87.1 ppm). This was confirmed by the X-ray data [Rh(1)···Cp, 1.7609(2) Å and Ir(1)···Cp, 1.7734(5) Å]. Consequently, the effective positive charge of the metal ion is decreasing, which is reflected in the Rh–N and Ir–N bond lengths with H_2_biim [Rh(1)–N(1), 2.114(2) Å; Rh(1)–N(3), 2.117(2) Å; Ir(1)–N(1), 2.109(4) Å; and Ir(1)–N(3), 2.105(4) Å]. This weaker coordination interaction of H_2_biim with the rhodium ion compared to the iridium ion was confirmed by the ^15^N NMR results (shift *δ*_Rh_ = − 173.8 ppm and shift *δ*_Ir_ = − 189.7 ppm). The chelating ligand H_2_biim is the same in both complexes; thus, *Δ*_coord._ = *δ*_complex_ − *δ*_ligand_ can be estimated according to the *δ*_Rh,Ir_ shift (the same is correct for the Cp ligand shift). It should be noted that the ligand H_2_biim is a tautomer under the experimental conditions, which effectively makes it impossible to measure the nitrogen spectrum and to determine the NH shift in the proton spectrum. Interestingly, by analysing the ^13^C NMR spectra of the rhodium complex, we observed a doublet around *δ* 95.4 ppm (*J*_Rh–C_ = 8.2 Hz). Moreover, the ^15^N NMR spectra of the same complex display a doublet centred ca. − 173.8 ppm, with a *J*_Rh–N_ value of 18.6 Hz, which is in the range typical for related compounds.

#### UV–visible spectroscopy

The UV–visible spectra of metal complexes **1**–**3** and H_2_biim (ligand) were determined in 1 × 10^−4^ M dry methanol at room temperature, and these spectra are presented in Figure S9. The Ru(II), Rh(III) and Ir(III) complexes are d^6^ low-spin metal complexes containing proper geometry-filled orbitals at the metal centres, which can interact with the ligand’s low lying π* orbitals, possibly resulting in metal-to-ligand charge transfer (MLCT) transitions (Table S3). The low energy absorption bands observed in the range from 330 to 410 nm are assigned to metal-to-ligand charge transition (MLCT) d*π*(M) to π*(L) transitions, while the high energy absorption bands observed in the range from 208 to 285 nm may be attributed to ligand-centre π–π*/*n*–π* transitions [39–42]. The pentamethylcyclopentadienyl (Cp) mainly shows low-intensity *π* → π* transition bands in the UV region overlapping with the biimidazole-based *π* → π*/*n* → π* transitions [43, 44].

#### FTIR spectroscopic study

The FTIR spectra of all the compounds exhibited strong bands in the region at 1527, 1531, and 1533 cm^−1^ and 1468, 1433, and 1458 cm^−1^, which corresponds to C=C and C=N stretching frequencies of the imidazole moieties in **1**, **2** and **3**, respectively. The N–H stretching frequency in the ligand (3144 cm^−1^) is also present in the same region in all the complexes (*ν* = 3145–3172 cm^−1^), indicating that deprotonation does not occur. Additionally, the metal complexes show sharp, characteristic peaks for the PF_6_^−^ counter ion at 849 cm^−1^ (**1**), 836 cm^−1^ (**2**) and 835 cm^−1^ (**3**) [45, 46]. Moreover, the IR spectra (Fig. S10) of the ruthenium complex show a broad absorption band at 3260 cm^−1^ for *ν*(O–H), which confirmed the presence of a water molecule in the complex structure. Additionally, vibrations appearing in the 2980–2700 cm^−1^ region are associated with aliphatic C–H stretching vibrations for the methyl and methine groups from the *p*-cymene moiety.

#### Stability studies in solution

**Fig. 5:**
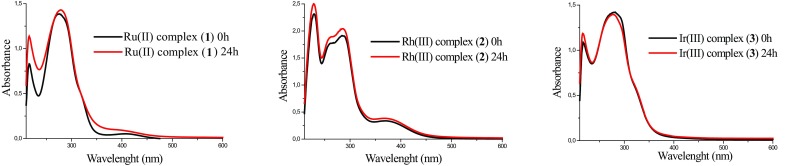
UV–Vis spectra of complexes at rt in buffer

Following the characterization of the complexes using standard spectroscopic methods before performing the biological studies, the stability of obtained complexes **1**–**3** was studied by UV–Vis and ^13^C NMR spectroscopy. To assess the stability of the complexes in aqueous solution, we selected Tris–HCl/NaCl buffer solution (pH 7.2, 298 K). The presence of a drop of DMSO ensured the solubility of the complexes. The spectra were unaltered over time, and no absorbance changes were observed in the UV–Vis spectra (Fig. 5), which indicated that the complexes were stable. Furthermore, stability experiments were performed on the complexes in DMSO-d_6_ by ^13^C NMR spectroscopy. The ^13^C NMR spectra of the analysed complexes showed no change over 24 h (Fig. S11). As a result, the stability studies suggest that the compounds have sufficient stability for the preparation of samples for biological assays. The significant stability of the complexes is probably due to the presence of strong hydrogen bonds and multiple noncovalent interactions (Fig. 4). The obtained compounds are inert towards substitution reactions due to the presence of strong bonding chelating ligand (H_2_biim) and inert metal ions [Rh(III), Ir(III)] which influenced compounds’ stability in solution significantly.

#### Antiproliferative activity and lipophilicity

**Fig. 6:**
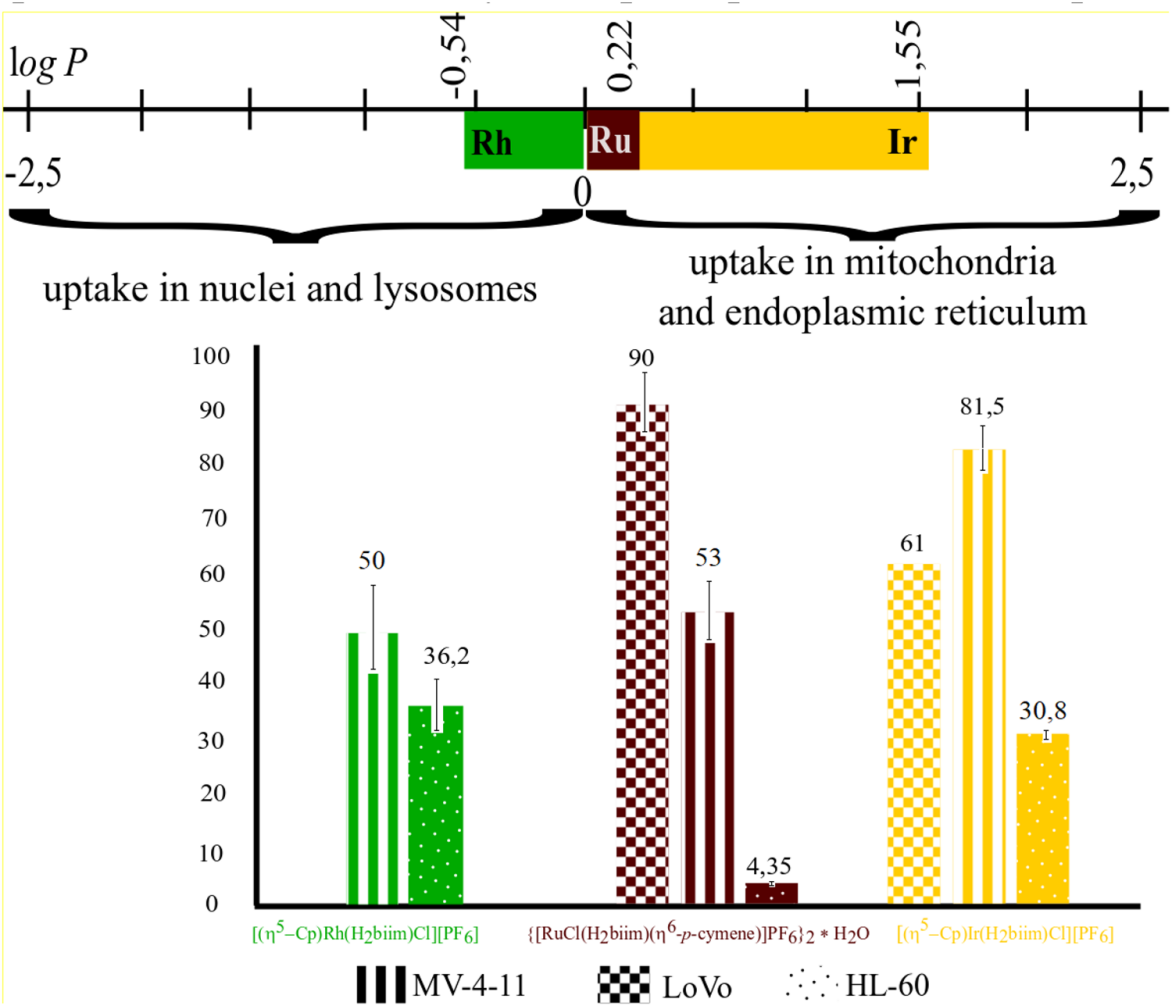
Comparison of lipophilicity, probably location in the cell and cytotoxic activity of analysed complexes

Lipophilicity is a very important physicochemical feature related to the pharmacokinetic behaviour of drug-like compounds [47]. Using the shake-flask technique, we obtained the log *P* value for **1** at +0.22 ± 0.01 and **2** at − 0.54 ± 0.02, whereas for **3**, the log *P* = + 1.55 ± 0.04, indicating that [(η^5^-Cp)RhCl(H_2_biim)]^+^ is more hydrophilic than {[RuCl(H_2_biim)(η^6^-*p*-cymene)]^+^ and [(η^5^-Cp)IrCl(H_2_biim)]^+^. Interestingly, in this case, the replacement of rhodium with a heavier homolog from the same group, iridium, in the obtained isostructural complex (**2** and **3**) led to significant differences in the lipophilicity (Fig. 6). Due to, the absorption and distribution of drug-like complexes with metal centres from the same group which accumulate in different cell components should influence on their biological activity. According to Horobin et al. [48, 49], a log *P* in the range from − 5 to 0 facilitates compounds crossing both cellular and nuclear membranes, so for rhodium compound **2** (log *P* = − 0.105) accumulation probably occurs mainly in the nuclei and lysosomes, whereas a log *P *> 0 suggests that the ruthenium (**1**) and iridium (**3**) compounds will specifically accumulate in the mitochondria and endoplasmic reticulum, but this requires further more detailed research. Taking this into consideration, the obtained complexes should possess different cytotoxic activities, which are mainly influenced by the type of metal centre ion in **1**, **2**, and **3** and the types of DNA/HSA interactions. The obtained unexpected results for the lipophilicity of the rhodium and iridium isostructural complexes were confirmed by multiple repetitions of the experiment.

The cell growth inhibition in the presence of complexes **1**–**3** was determined using MTT and SRB assays after a 96 h incubation period against a panel of four human tumour cell lines, including LoVo, HL-60, MV-4-11, and MCF-7 cells, as well as the BALB/3T3 healthy mouse cell line (Table 3). Notably, the obtained complexes show no cytotoxic effects towards the normal BALB/3T3 cell line compared to cisplatin (IC_50_ = 2.20 µg). Importantly, {[RuCl(H_2_biim)(η^6^-*p*-cymene)]PF_6_}_2_·H_2_O (**1**) displayed the highest activity against HL-60 cells (IC_50_ 4.35 µg), while the IC_50_ values of Rh(III) and Ir(III) were 36.2 µg and 30.86 µg, respectively. According to the log *P* values (log *P* > 0), the ruthenium and iridium complexes are active against the same cancer cell lines (LoVo, HL-60, and MV-4-11), whereas the rhodium complex (log *P* < 0) was only active against HL-60 and MV-4-11. All the compounds were not only inactive against the normal cell line at the tested concentration range but also against the MCF-7 cell line. These results suggest the cytotoxic selectivity of the obtained organometallic complexes. It should be underlined that the biological potencies of two isostructural half-sandwich rhodium and iridium complexes (metals from the same group) in the same overall ligand set are not the same. Studying similarities and differences in biological activity of investigated analogues, it should be pointed out that the rhodium complex is not active against MCF-7 and LoVo cells, while the iridium compound is only not active against the MCF-7 cell line. Moreover, the rhodium complex is approximately twofold more active against MV-4-11 but slightly less active against the HL-60 cell line compared with the iridium complex. The differences in the biological activity of potential drugs, such as discussed complexes, are most likely derived from their absorption and distribution into different cell components, depending on their lipophilicity (log *P*_Rh_ = − 0.54, log *P*_Ir_ = + 1.55). Similar differences in anticancer activities in vitro and in vivo have been discussed by Sadler et al. [9–11] for ruthenium(II) (RM175) and its osmium(II) analogue (AFAP51).

**Table 3:** IC_50_ values representing the antiproliferative activity of analysed complexes in panel of four human cancer cell lines and normal mice fibroblasts compared to cisplatin

Compound	IC_50_ (μg)				
	MV-4-11	HL-60	MCF-7	LoVo	BALB/3T3
{[RuCl(H_2_biim)(η^6^-*p*-cymene)]PF_6_}_2_·H_2_O	53 ± 9.9	4.35 ± 0.15	> 100	90 ± 11	> 100
[(η^5^-Cp)RhCl(H_2_biim)]PF_6_	50 ± 13	36.2 ± 7.7	> 100	> 100	> 100
[(η^5^-Cp)IrCl(H_2_biim)]PF_6_	81.5 ± 9.4	30.86 ± 0.75	> 100	61 ± 0	> 100
H_2_biim	> 100	> 100	> 100	> 100	> 100
Cisplatin	0.55 ± 0.15	0.25 ± 0.12	2.17 ± 0.55	1.96 ± 0.68	2.20 ± 0.43

#### Interactions with biomolecules

Organometallic complexes act as potential drugs by interacting with various targets including DNA and proteins, which is probably the explanation for their anticancer activities [50, 51]. Taking this into account and our previously published anticancer results [16–18], we evaluated the potential interactions between CT-DNA, HSA and GSH with the obtained ruthenium(II) (**1**), rhodium(III) (**2**) and iridium(III) (**3**) complexes.

**Fig. 7:**
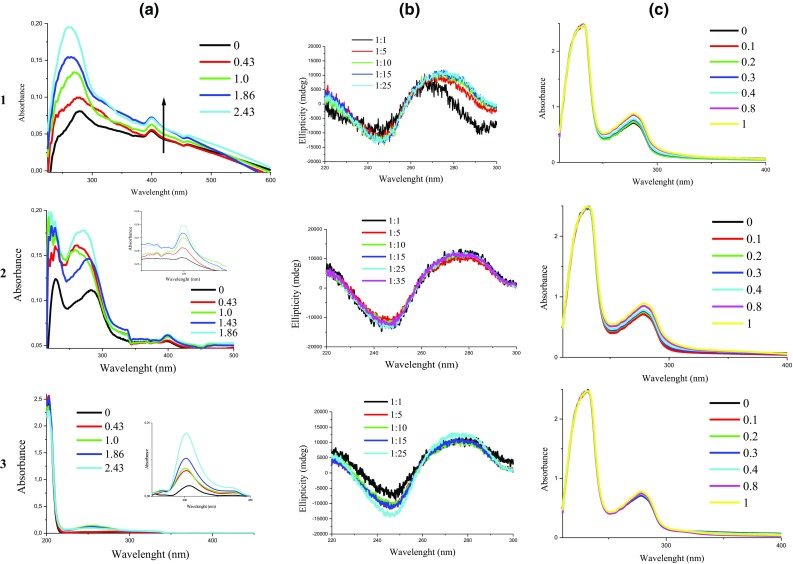
Spectra of **1**, **2** and **3** complexes interactions with: **a** CT-DNA–UV–Vis spectra (7 µM complex: 0.0, 3, 7, 13, 17 µM of CT-DNA) (zoom portion of figure shows clearly the existence of changes); **b** CT-DNA–CD spectra (CT-DNA 300 µM) after 24 h incubation at different [complex]/[DNA] ratios at 37 °C; **c** HSA in the presence of different concentrations of complex

The interactions of **1**, **2**, and **3** with CT-DNA were studied by UV–Vis and CD spectroscopy to investigate the possible DNA-binding modes and to determine the binding constants (*K*_b_). The UV–Vis spectra shown in Fig. 7a display the maximum absorption of {[RuCl(H_2_biim)(η^6^-*p*-cymene)]PF_6_}_2_·H_2_O at 401 nm and [(η^5^-Cp)RhCl(H_2_biim)]PF_6_ at 398 nm; moreover, a hyperchromic effect with a red shift of approximately 1 nm was observed upon the addition of CT-DNA. The [(η^5^-Cp)IrCl(H_2_biim)]PF_6_ complex exhibited hyperchromism at 401 nm but with a blue shift of approximately 3 nm. These spectral characteristics (hyperchromic effect) suggest that the complexes interact with CT-DNA through non-intercalative binding modes such as outside binding. This may be due to electrostatic interactions between the positively charged [RuCl(H_2_biim)(η^6^-*p*-cymene)]^+^, [(η^5^-Cp)IrCl(H_2_biim)]^+^ and [(η^5^-Cp)IrCl(H_2_biim)]^+^ complex units and the negatively charged phosphate backbone at the periphery of the double-helix CT-DNA [16, 52]. These data are in agreement with previous reports in the literature [53] and with previous data obtained by us [16]. The magnitude of the binding strengths of the compounds with CT-DNA can be evaluated via their intrinsic binding constant K_b_. The K_b_ values are equal 2.5 × 10^5^ M^−1^, 5 × 10^4^ M^−1^, and 5 × 10^4^ M^−1^ for the ruthenium (**1**), rhodium (**2**) and iridium (**3**) complexes, respectively. The presented data demonstrated that all the complexes distinctly interact with CT-DNA and that complex **1** binds the most efficiently.

The circular dichroism spectra were adopted to confirm our results obtained by UV–Vis. The changes in the CD signals of the DNA observed upon interaction with drugs may often be assigned to corresponding changes in DNA structure. The observed CD spectrum of calf thymus DNA consists of a positive band at 275 nm (UV: *λ*_max_, 260 nm) due to base stacking and a negative band at 245 nm due to helicity, which is characteristic of DNA in the right-handed B form [54, 55]. The CD spectra obtained for CT-DNA and **1**, **2**, and **3** in different DNA:complex ratios (1:1, 5:1, 10:1, 15:1, 25:1) after 24 h (Fig. 7b) of incubation imply a non-intercalative mode of interaction between the DNA and complexes. The DNA region of the spectrum from 210 to 300 nm revealed changes in the DNA conformation with a positive peak at approximately 278 nm showing increased ellipticity and a negative band (~ 248 nm) showing a tendency for continuously decreased ellipticity with increasing concentrations of the **1**, **2**, and **3** complexes (Fig. 7). Additionally, the maximum absorption peak for Ru(II) at 268 nm was accompanied by a red shift (ca. 8 nm), while that the peak intensity for Ir(III) at 278 nm was changed with a blue shift of approximately 2 nm, which suggests that the ruthenium complex interacts with CT-DNA much more extensively. CD spectral analysis of the Rh(III) complex upon the addition of CT-DNA did not suggest significant changes in the band at 278 nm and was not accompanied by a wavelength shift. According to the literature data, little or no perturbation in the base stacking and helicity bands suggests simple groove binding and electrostatic interactions with the complex [56].

Due to albumin’s remarkable binding properties and high abundance in blood plasma, serum plays a crucial role in the drug delivery system [57]. To understand the nature of its binding with the complexes, the absorption intensity of human serum albumin (HSA) has been investigated in the presence of the complexes under study. Absorbance titration studies have been performed at room temperature using HSA (0.5 μM) with varying concentrations of **1**–**3** (0–50 μM) in the range of 290–500 nm (*λ*_max_ 278 nm). From the spectra (Fig. 7c), it is clear that the absorption intensity of HSA band increases without any shift in the presence of the ruthenium, rhodium and iridium complexes indicating a static interaction between HSA and the complexes. Using the graph of (*ε*_a_ − *ε*_f_) versus [complex], the binding constants were calculated as 1.5 × 10^4^ (**1**), 5 × 10^4^ (**2**), and 1 × 10^4^ (**3**). These values are within the range of 10^4^ to 10^6^ M^−1^, as expected for good HSA carrier activity in vivo [58]. To gain a better understanding in the conformational behaviour of HSA in the albumin: ruthenium/rhodium or iridium complex, CD spectroscopic measurements were performed. The CD spectra of HSA at pH 7.2 exhibited two negative minima at 208 and 222 nm, which is characteristic of an α-helical protein structure. Since the α-helix is just one secondary structure element, the structural changes in albumin could be evaluated by the α-helical structure content [58]. Figure S12 shows the CD spectra of native HSA and HSA with increasing concentrations of complexes. As shown, the ellipticity values were found to increase in the presence of complexes **1**–**3**, which indicated the loss of protein α-helical structure. The changes in the helicity of HSA indicate that the chains keep unfolding in the presence of different concentrations of the complexes (Fig. S12).

The literature data demonstrated that glutathione (γ-glutamylcysteinylglycine, GSH) containing (SH) groups act as enzyme cofactors, antioxidants and antitoxins. Therefore, GSH has various physiological functions and is the most abundant nonprotein thiol in mammalian cells [59]. It was presented that the cytotoxicity of some organometallic anticancer compounds [60] is derived from the fact that the complex acts as a catalyst for the oxidation of GSH to GSSG, which simultaneously causes an increase in ROS levels, pointing to the cytotoxic activities of complexes. Simultaneously, GSH can coordinate with the metals in anticancer drugs to form less toxic GSH conjugates, thereby detoxifying anticancer drugs. According to our UV–Vis studies, rhodium complex (**2**) is the most GSH reactive while ruthenium complex **1** is less active and its activity is similar to cisplatin (Table 4). Therefore, low affinity of the complex to GSH might have a benefit in potential therapy.

**Table 4:** Concentration of unreacted GSH after 30 h incubation with complexes

Complex	Concentration of unreacted GSH (mM) GSH_0_ = 0.05 mM
**1**	0.035 ± 0.001
**2**	0.018 ± 0.001
**3**	0.027 ± 0.001
**Cisplatin**	0.034 ± 0.001

Moreover, to examined products of the GSH reaction with the compounds the ESI mass spectra of free GSH, GSH-cisplatin, GSH-complexes (**1**–**3**) were recorded (Fig. S13). The detailed information of several interaction products ions extracted from reaction solution is given in Table 5. The results show that ruthenium (**1**), rhodium (**2**) and iridium (**3**) complexes formed adducts with GSH, with fragments of GSH or fragments of GSSG (Table 5, Fig. S13a–c).

**Table 5:** Assignment of (+) mass ions in the ESI mass spectra shown in Fig. S13 compared to calculated mass ions

*m/z*	Assignment	Calcd.	*m/z*	Assignment	Calcd.
{[RuCl(H_2_biim)(η^6^-*p*-cymene)]PF_6_}_2_·H_2_O			[(η^5^-Cp)RhCl(H_2_biim)]PF_6_		
288.23	[RuCl(H_2_biim)H_2_O]^+^	288.93	371.00	[(η^5^-Cp)Rh(Hbiim)]^+^	371.07
368.99	[Ru(Hbiim)(η^6^-*p*-cymene)]^+^	368.06	308.04	[GSH+H]^+^	308.09
404.97	[RuCl(H_2_biim)(η^6^-*p*-cymene)]^+^	404.03	406.97	[(η^5^-Cp)RhCl(H_2_biim)]^+^	407.05
676.02	[Ru(H_2_biim)(η^6^-*p*-cymene)+GS]^+^	676.19	544.00	[(η^5^-Cp)RhCl(H_2_biim)PF_6_+Na]^+^	544.03
808.92	[Ru(H_2_biim)(η^6^-*p*-cymene)+GSSG^a^]^+^	808.18	678.03	[(η^5^-Cp)Rh(H_2_biim)+GS]^+^	678.16
			698.01	[(η^5^-Cp)Rh(H_2_biim)+GSSG^b^]^+^	698.15
[(η^5^-Cp)IrCl(H_2_biim)]PF_6_			Cisplatin		
308.04	[GSH+H]^+^	308.09	288.23	[PtClNH_3_CH_3_CN]^+^	287.99
461.05	[(η^5^-Cp)Ir(Hbiim)]^+^	461.13	308.03	[GSH+H]^+^	308.09
497.01	[(η^5^-Cp)IrCl(H_2_biim)]^+^	497.10	353.19	[GSH+2Na]^+^	353.06
549.66	[(η^5^-Cp)Ir(H_2_biim)+GS^a^]^+^	549.13	381.22	[Pt+GS^c^]^+^	381.01
563.68	[(η^5^-Cp)Ir(H_2_biim)+GS^b^]^+^	563.13	554.45	[PtClNH_3_+GSH]^+^	554.04
768.08	[(η^5^-Cp)Ir(H_2_biim)+GS]^+^	768.21	711.44	[PtCl+GSSG^c^]^+^	711.03
GSH					
201.94	[GSH^d^+K]^+^	201.99	569.29	[GSSG-CO_2_+H]^+^	569.17
409.10	[GSSG^d^-H]^+^	409.05	585.23	[GSSG-CO+H]^+^	585.16
531.31	[GSSG^e^+CH_3_CN+Na]^+^	531.11	779.60	[3GSH-2CO_2_-2CO+2H]^+^	779.30
553.29	[GSSG^f^+CH_3_CN+H]^+^	553.14			

GSH (glutathione) = C_10_H_17_N_3_O_6_S; GSH (glutathione fragments) = ^a^C_3_H_3_OS; ^b^C_3_H_4_NOS; ^c^C_7_H_10_N_2_O_2_S; ^d^C_5_H_9_NO_3_S; GSSG (glutathione disulfide) = C_20_H_32_N_6_O_12_S_2_; GSSG (glutathione disulfide fragments) = ^a^C_14_H_25_N_5_O_7_S_2_; ^b^C_9_H_18_N_4_O_5_S_2_; ^c^C_15_H_23_N_5_O_9_S_2_; ^d^C_12_H_16_N_4_O_8_S_2_; ^e^C_15_H_23_N_4_O_9_S_2_; ^f^C_16_H_26_N_5_O_10_S_2_

## Conclusion

In conclusion, new d^6^ organometallic ruthenium (**1**), rhodium (**2**) and iridium (**3**) complexes containing 2,2′-biimidazole (H_2_biim) as a chelating ligand were prepared and characterized. Crystallographic studies showed that the piano-stool complex **1** possesses a dimeric structure due to strong hydrogen bonds between the cationic species. In contrast, the asymmetric units in complexes **2** and **3** consist of cationic complexes in a monomeric form. The obtained compounds possess significant solution stability, which is probably achieved due to the presence of strong hydrogen bonds and multiple noncovalent interactions (especially for **1**). It should be underlined that the chelating ligand (H_2_biim) and the inertness of the metal ions [Rh(III), Ir(III)] significantly influenced compounds’ stability in solution. Similar to the osmium and ruthenium complex analogues investigated by Sadler’s group, the biological potencies of the two obtained isostructural half-sandwich rhodium and iridium complexes (metals from the same group) are not the same. The differences in complexes **2** and **3** are potentially derived from their absorption and distribution into different cell components, which is based on their lipophilicity (log *P*_Rh_ = − 0.54, log *P*_Ir_ = + 1.55). A comparison of the cytotoxicity showed that the rhodium complex is not active against MCF-7 and LoVo cells, while the iridium compound is only not active against the MCF-7 cell line. Moreover, the rhodium complex is approximately twofold more active against MV-4-11 cells but slightly less active against the HL-60 cell line compared with the iridium complex. Importantly, {[RuCl(H_2_biim)(η^6^-*p*-cymene)]PF_6_}_2_·H_2_O (**1**) displayed the highest activity against HL-60 cells (IC_50_ 4.35 µg). Additionally, it should be emphasized that the successfully obtained complexes were inactive against the normal cell line. UV–Vis and CD data suggest that the compounds can efficiently bind to protein targets (HSA) and interact with calf thymus DNA. The spectral characteristics (hyperchromic effect) suggest that the complexes interact with CT-DNA through electrostatic interactions between the positively charged [RuCl(H_2_biim)(η^6^-*p*-cymene)]^+^, [(η^5^-Cp)IrCl(H_2_biim)]^+^ and [(η^5^-Cp)IrCl(H_2_biim)]^+^ complex units and the negatively charged phosphate backbone at the periphery of the DNA double-helix. Moreover, we studied the interactions of the analysed complexes with GSH. The ESI mass spectra results confirmed that **1**, **2** and **3** obtained complexes bound to GSH and formed adducts with GSH, with fragments of GSH or GSSG. Additionally, the UV–Vis results demonstrated that ruthenium complex **1** is considerably less active in the reaction with GSH than the rhodium (**2**) and iridium (**3**) complexes. Simultaneously, the ruthenium(II) dimer displays better cytotoxicity compared to complexes **2** and **3**.

## Electronic supplementary material

Below is the link to the electronic supplementary material.

Supplementary material 1 (PDF 1779 kb)

Supplementary material 2 (PDF 143 kb)

Supplementary material 3 (PDF 139 kb)

Supplementary material 4 (PDF 141 kb)
