# Supplementary material for: Similarities and differences in d6 low-spin ruthenium, rhodium and iridium half-sandwich complexes: synthesis, structure, cytotoxicity and interaction with biological targets
Source: J Biol Inorg Chem. 2019 May 21;24(4):591–606. doi: 10.1007/s00775-019-01665-2 (PMC6598960; doi:10.1007/s00775-019-01665-2)
Supplement: Supplementary file 1 — Supplementary material 1 (PDF 1779 kb) [file 775_2019_1665_MOESM1_ESM.pdf]

Electronic Supplementary Material (ESM)

Similarities and differences in d^6 low spin ruthenium, rhodium and iridium half-sandwich complexes: Synthesis, structure, cytotoxicity and interaction with biological targets

Agnieszka Gilewska, Barbara Barszcz, Joanna Masternak, Katarzyna Kazimierczuk, Jerzy Sitkowski, Joanna Wietrzyk, Eliza Turlej

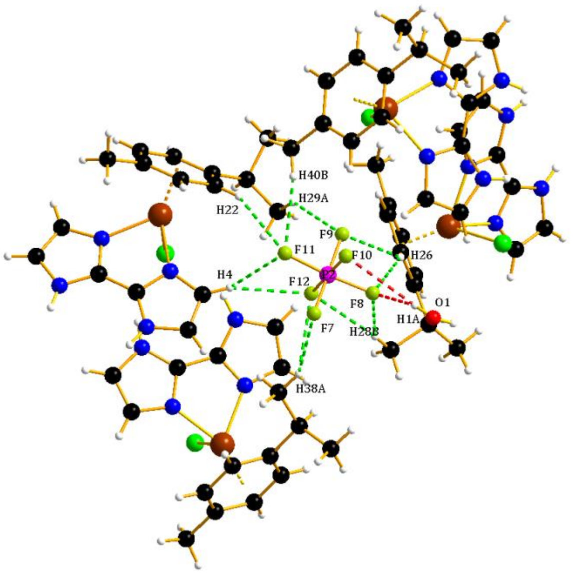

Fig. S1. The C-H···F and O-H···F interactions in crystal structure of complex **1**.

The crystal packing of complex **1** shows some intermolecular interactions, including N-H···O, N/C/-H···F, C-H···π and π···π, that contribute to the stability of the compound. More specifically, one protonated N-H group from the 2,2'-biimidazole ligand coordinating to Ru(1) forms trifurcated hydrogen bonds, including N(1)-H(1)···O(1), N(1)-H(1)···F(5) and N(1)-H(1) ···F(6). The second N-H group in the same ligand is a donor of a proton in the N(1')-H(1')···O(1) bond with the water molecule present in the compound structure. At the same time, one protonated N-H group from the 2,2'-biimidazole ligand coordinating to Ru(2) is bifurcated and forms a combination of a hydrogen bond with the chloride ion (N(11)-H(11)···Cl(1)) and PF_6^- anion (N(11)-H(11)···F(2)). The second protonated N-H group participates in the formation of one hydrogen bond, N(11')-H(11')···Cl(1). In the crystal structure of **1**, the water molecule forms two bifurcated hydrogen bonds, O(1)-H(1A)···F(8)/F(10) and O(1)-H(1B)···F(4)/F(6), with the PF_6^- counterion (Fig. 2b, Fig. S1).

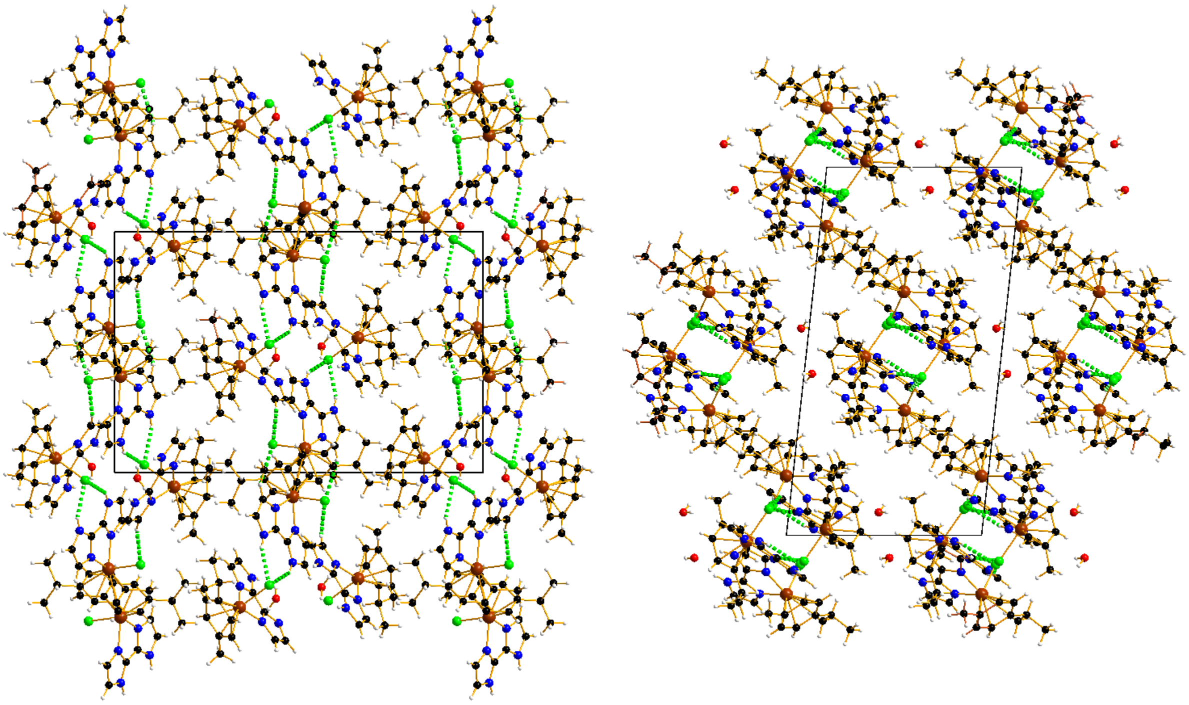

a) b)

Fig. S2. a) Part of an infinite chain of molecules arising through H-bond interactions in complex **1**, b) crystal packing with water molecules between tetrameric units (counterions are omitted for clarity of the structure).

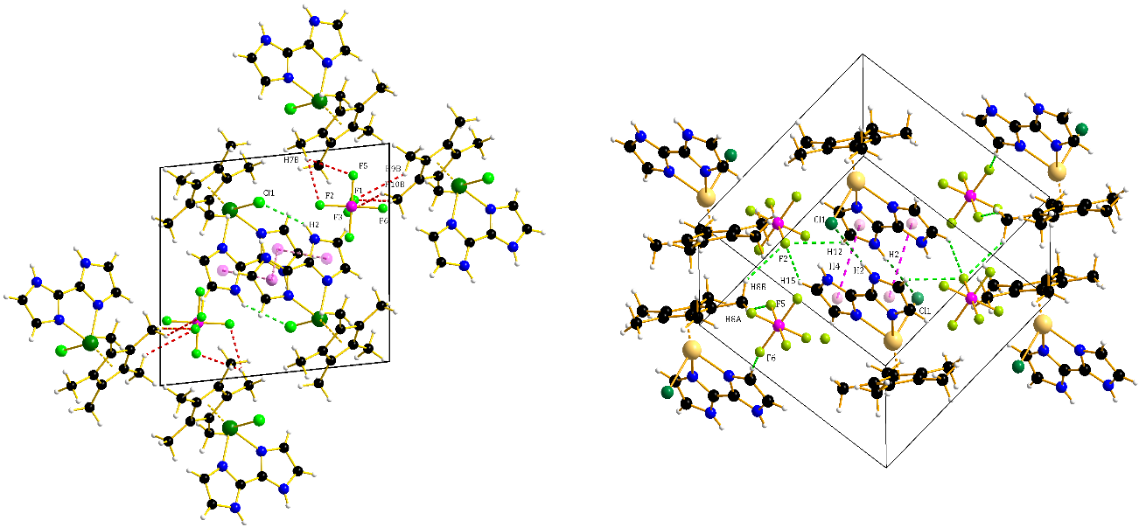

Fig. S3. Crystal structures of **2** (left)) and **3** (right) with marked N-H···Cl, C-H···F and π···π interactions.

Table S1. Crystal data and structure refinement for **1-3** complexes.

Empirical formula	$C_{32}H_{42}Cl_2F_{12}N_8OP_2Ru_2$	$C_{16}H_{21}ClF_6N_4PRh$	$C_{16}H_{21}ClF_6N_4PIr$
Formula weight	1117.71	552.70	641.99
Temperature [K]	120(2)	120(2)	120(2)
Wavelength (Mo K_{α}) [Å]	0.71073	0.71073	0.71073
Crystal system, space group	Monoclinic, P $2_1/n$	Triclinic, *P*-1	Triclinic, *P*-1
a [Å] / α [°]	12.0477(8) /90	7.9505(2) / 95.068(2)	7.990(3) / 95.04(2)
b [Å] / β [°]	14.8587(7) / 96.246(5)	11.0726(3) / 95.190(2)	11.140(3) / 95.47(2)
		11.5662(3) / 99.370(2)	11.537(3) / 100.15(2)

c [Å] / γ [°]	22.9202(15) / 90		
Volume [A^3]	4078.7(4)	998.33(5)	1000.5(5)
Z, calculated density [Mg/m^3]	4, 1.820	2, 1.844	2, 2.131
μ [mm^{-1}]	1.045	1.138	6.951
F(000)	2232	552	616
θ range [°]	2.429 to 25.499	2.445 to 25.498	2.441 to 25.497
Limiting indices	-14<=h<=14, -15<=k<=18, -27<=l<=27	-9<=h<=9, -13<=k<=13, -14<=l<=14	-9<=h<=9, -13<=k<=13, -13<=l<=13
Reflections collected/unique	17084 / 7473 [R(int) = 0.0500]	10832 / 3696 [R(int) = 0.0248]	10981 / 3717 [R(int) = 0.0417]
Completeness to θ_{max} /%	98.6	99.9	99.9
Refinement method		Full-matrix least-squares on F^2	
Data / restraints / parameters	7473 / 0 / 538	3697 / 0 / 267	3717 / 0 / 267
Goodness-of-fit on F_2	1.143	1.076	0.999
Final R indices [I>2sigma(I)]	R_1 = 0.0730, wR_2 = 0.2073	R_1 = 0.0289, wR_2 = 0.0746	R_1 = 0.0268, wR_2 = 0.0668
R indices (all data)	R_1 = 0.0881, wR_2 = 0.2301	R_1 = 0.0298, wR_2 = 0.0752	R_1 = 0.0285, wR_2 = 0.0674
Largest diff. peak and hole [$e.A^{-3}$]	2.116;-1.044	1.568;-0.643	1.332; -1.286

Table S2. Selected hydrogen bonding parameters for **1**-**3** complexes.

Complexes	D-H···A	d(D-H)	d(H···A)	d(D···A)	∢(DHA)	Symmetry code
**1**	N(1)-H(1)···O(1)	0.86	2.0959(85)	2.8513(12)	146.35	
	N(1')-H(1')···O(1)	0.86	2.1715(88)	2.9095(12)	143.72	
	N(11)-H(11)···Cl(1)	0.86	2.3691(24)	3.1579(93)	153.83	
	N(11')-H(11')···Cl(1)	0.86	2.4691(23)	3.2442(86)	150.25	
	N(11)-H(11)···F(2)	0.86	2.8235(84)	3.1416(13)	103.82	
	C(15)-H(15)···F(2)	0.93	2.5122(98)	3.0060(14)	113.50	
	C(29)-H(29B)···F(2)	0.96	2.6320(11)	3.2209(16)	119.99	
	C(27)-H(27)···F(2)	0.98	2.8634(11)	3.5163(15)	124.81	
	C(27)-H(27)···F(6)	0.98	2.7505(74)	3.4119(14)	125.28	
	O(1)-H(1A)···F(8)	0.85	1.9167(10)	2.7903(14)	162.67	
	O(1)-H(1A)···F(10)	0.85	2.8111(10)	3.5061(14)	140.07	
	O(1)-H(1B)···F(6)^{i}	0.85	2.5582(72)	3.1732(12)	130.20	^{i}1+x, y, z
	O(1)-H(1B)···F(4)^{i}	0.85	2.0045(98)	2.8442(13)	169.79	
	N(1)-H(1)···F(6)^{i}	0.86	2.6442(74)	3.2136(14)	124.85	
	N(1)-H(1)···F(5)^{i}	0.86	2.5907(98)	3.0549(12)	115.00	
	C(5)-H(5)···F(5)^{i}	0.93	2.7619(80)	3.1378(14)	105.23	
	C(32)-H(32)···F(5)^{i}	0.93	2.6675(10)	3.3430(16)	130.08	
	C(32)-H(32)···F(1)^{i}	0.93	2.6313(11)	3.5336(16)	163.63	
	C(33)-H(33)···F(3)^{i}	0.93	2.8177(75)	3.5133(14)	132.44	
	C(38)^{ii}-H(38C)^{ii}···F(12)	0.96	2.5916(10)	3.4348(17)	146.62	^{ii}1.5-x, 0.5+y, 0.5-z
	C(38)^{ii}-H(38C)^{ii}···F(7)	0.96	2.7360(86)	3.4203(16)	128.73	
	C(4)^{ii}-H(4)^{ii}···F(7)	0.93	2.6822(92)	3.5768(13)	161.32	
	C(4)^{ii}-H(4)^{ii}···F(11)	0.93	2.5807(91)	3.3079(13)	135.29	
	C(22)^{ii}-H(22)^{ii}···F(11)	0.93	2.8435(11)	3.7036(15)	154.16	
	C(40)^{iii}-H(40B)^{iii}···F(11)	0.96	2.6152(99)	3.4717(16)	148.78	^{iii}2-x, 1-y, 1-z
	C(29)^{ii}-H(29A)^{ii}···F(9)	0.96	2.7593(82)	3.5363(14)	138.52	
	C(26)^{iv}-H(26)^{iv}···F(9)	0.93	2.6479(88)	3.2712(13)	124.98	^{iv}1+x, y, z
	C(26)^{iv}-H(26)^{iv}···F(9)	0.93	2.5914(12)	3.4449(16)	152.74	

	C(28)^{iv}-H(28)^{iv}···F(8)	0.96	2.7876(10)	3.6415(17)	148.68	
	C(28)^{iv}-H(28)^{iv}···F(7)	0.96	2.8576(10)	3.7851(17)	162.89	
	C(38)-H(38C)···Cg	0.96	3.0303(2)	3.8288(15)	141.51	
	Cg···Cg			3.7835(2)		
**2**	C(9)^{ii}-H(9B)^{ii}···F(1)^{i}	0.99	2.8573(26)	3.5721(45)	130.45	^{i}1+x, y, z<br>^{ii}x, y, -1+z
	C(10)^{ii}-H(10C)^{ii}···F(1)^{i}	0.98	2.4265(23)	3.3283(39)	152.76	
	C(7)^{iii}-H(7B)^{iii}···F(2)^{i}	0.98	2.4742(33)	3.4138(47)	160.50	^{iii}2-x, 2-y, 1-z
	C(10)^{ii}-H(10B)^{ii}···F(3)^{i}	0.99	2.6281(25)	3.4439(43)	140.88	
	C(6)^{ii}-H(6B)^{ii}···F(6)^{i}	0.98	2.6957(27)	3.2181(44)	113.77	
	C(7)^{i}-H(7B)^{i}···F(5)^{i}	0.98	2.6658(32)	3.4638(46)	148.78	
	N(2)^{iv}-H(2)^{iv}···Cl(1)	0.88	2.5809(60)	3.3587(22)	147.90	^{iv}2-x, 1-y, 1-z
	Cg···Cg^{iv}			3.5101(1)		
	Cg···Cg^{v}			3.6339(1)		^{v}1-x, 1-y, 1-z
**3**	C(16)^{i}-H(16)^{i}···F(6)	0.95	2.4849(35)	3.4121(53)	165.17	^{i}1-x, 1-y,2-z
	C(8)^{i}-H(8C)^{i}···F(5)	0.98	2.4318(33)	3.3301(57)	152.24	
	C(15)-H(15)···F(2)^{ii}	0.95	2.4127(39)	3.2038(60)	140.59	^{ii}1+x, y, z
	C(12)^{iii}-H(12)^{iii}···F(2)^{ii}	0.95	2.7302(43)	3.1969(63)	111.05	^{iii}1-x, 1-y, 1-z
	C(8)^{i}-H(8B)^{i}···F(2)^{ii}	0.98	2.6976(36)	3.4924(61)	138.45	
	N(2)-H(2)···Cl(1)^{iii}	0.88	2.3936(12)	3.1956(38)	151.58	
	Cg···Cg^{iii}			3.5287(9)		

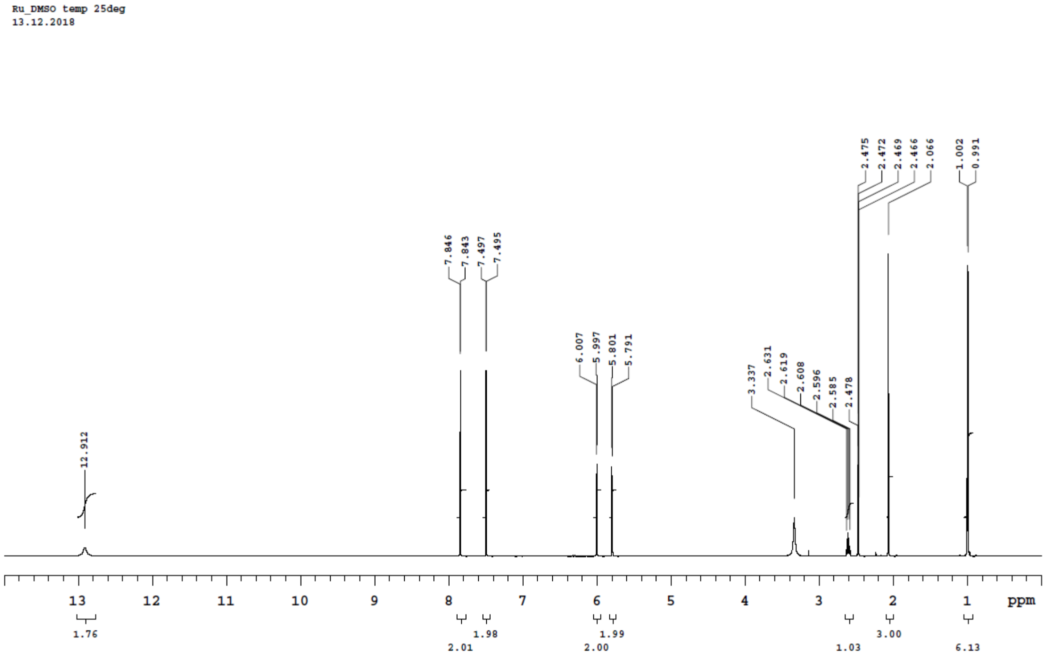

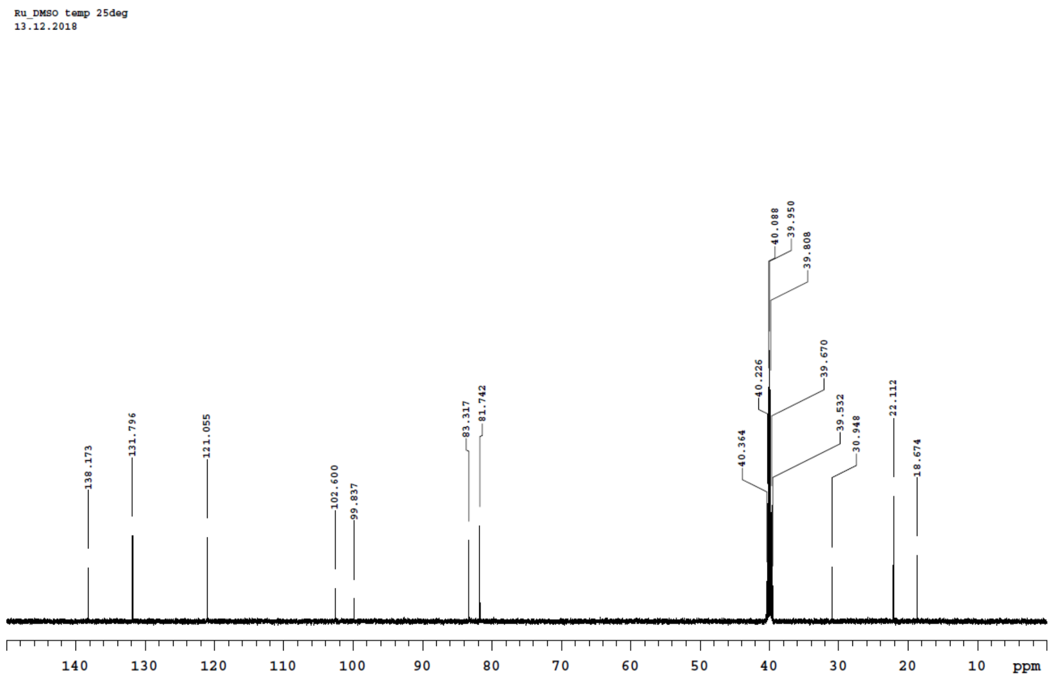

Fig. S5. ^{1}H and ^{13}C NMR spectra of complex **1** in DMSO-d_6

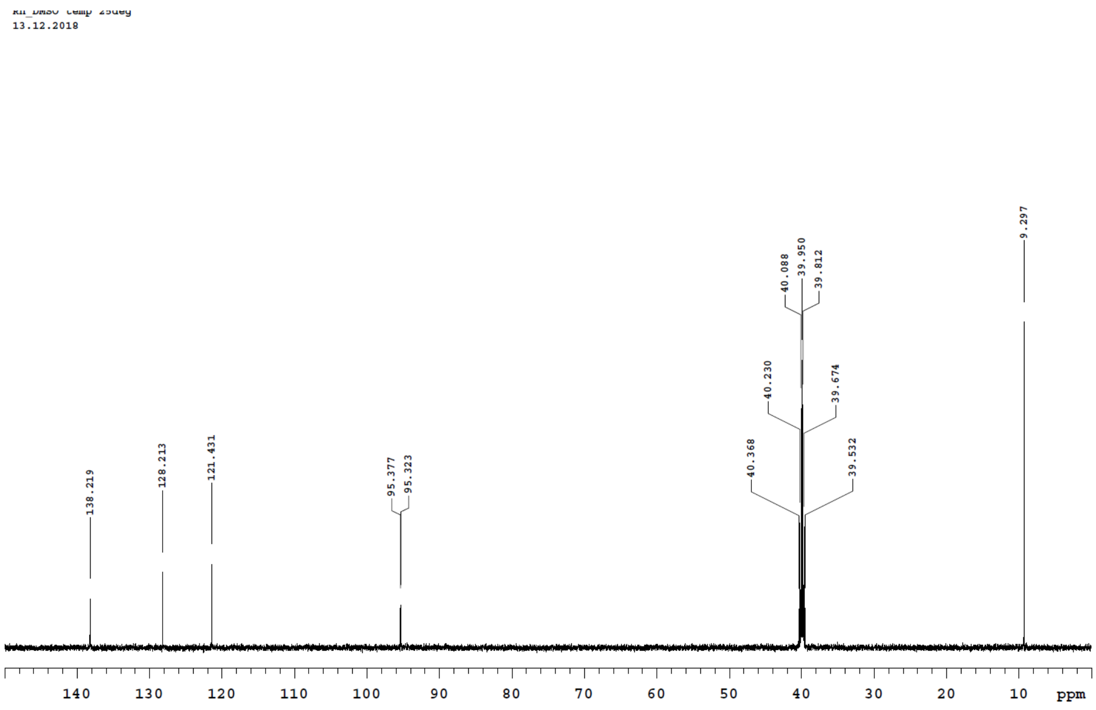

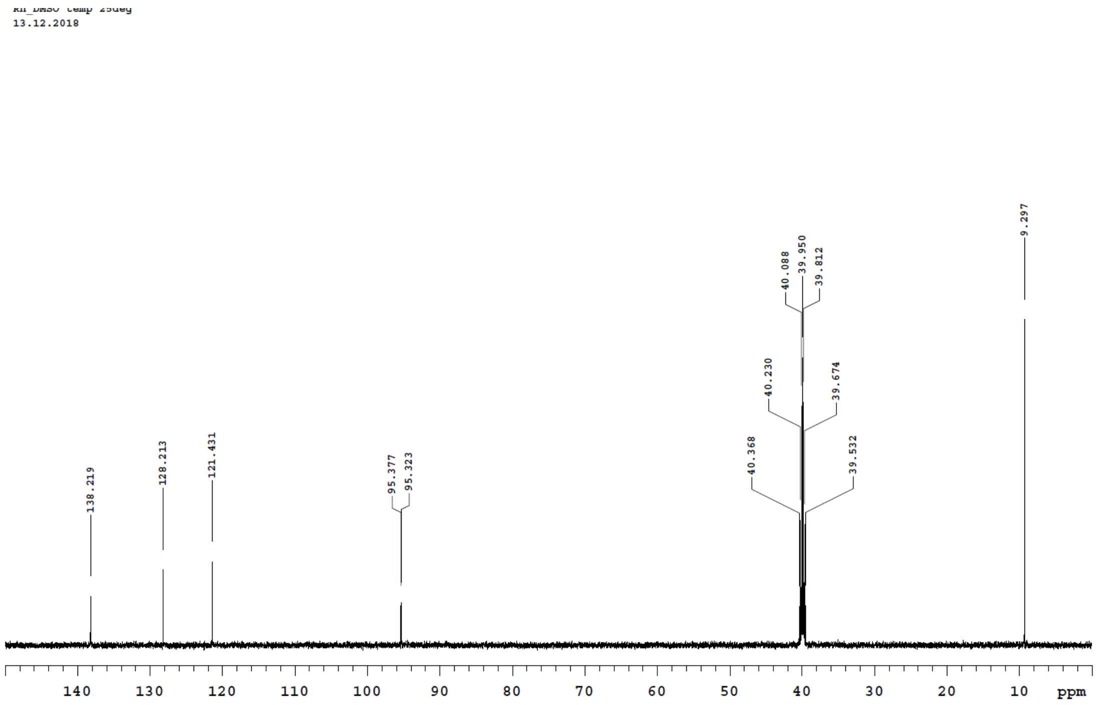

Fig. S6. ^{1}H and ^{13}C NMR spectra of complex **2** in DMSO-d_6

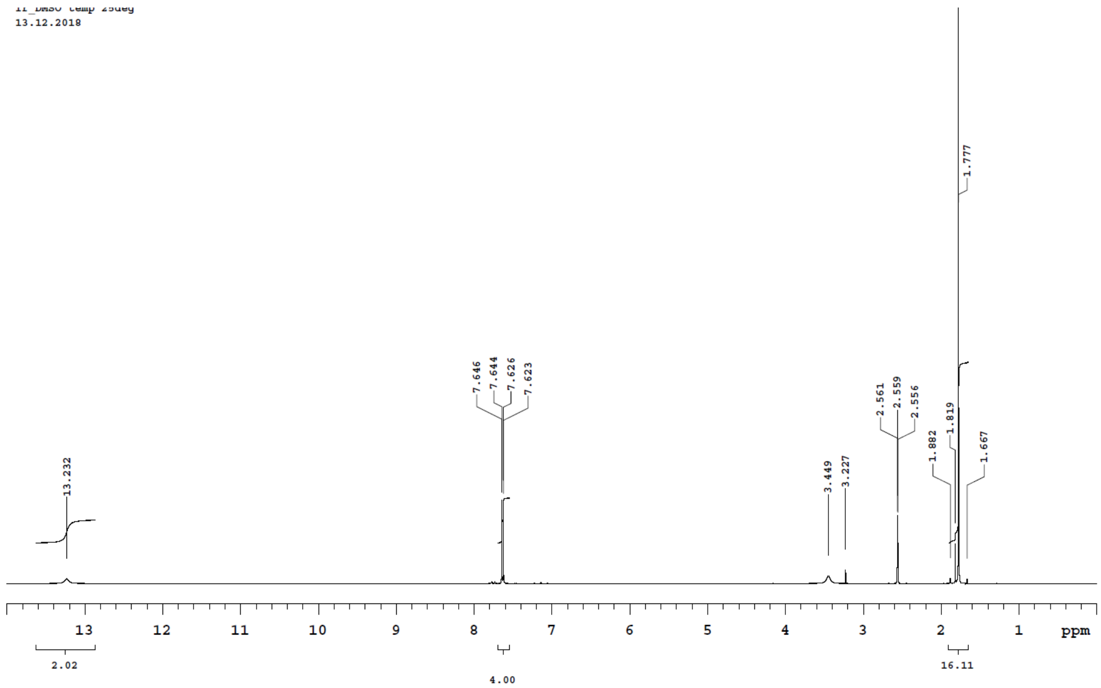

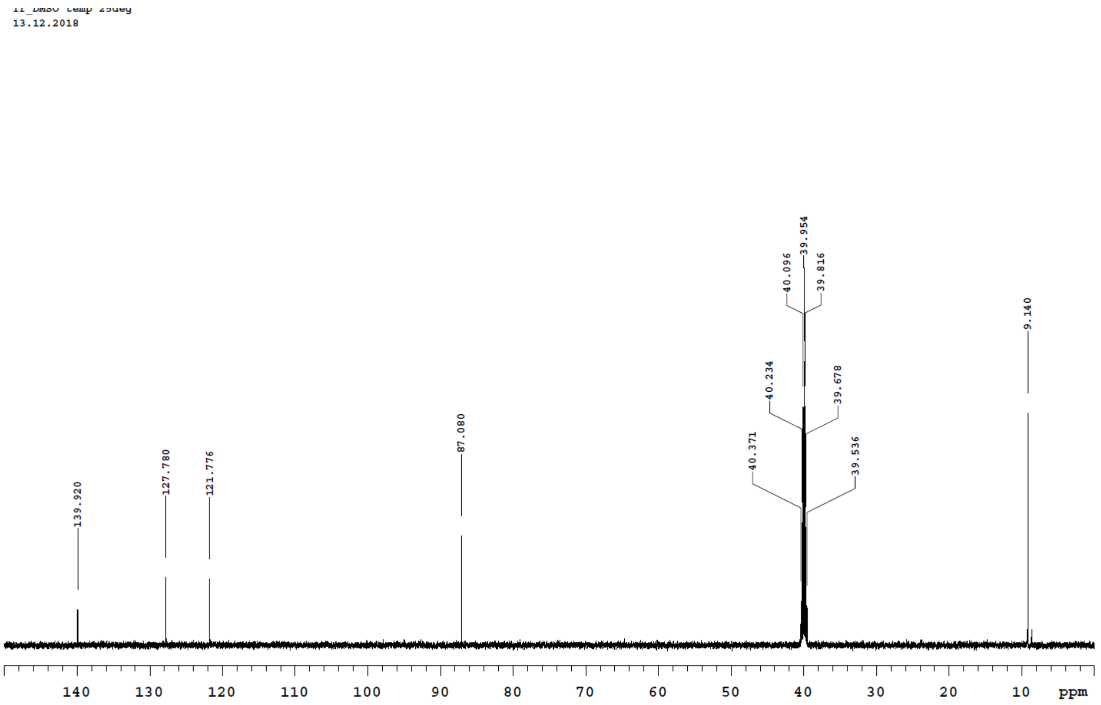

Fig. S7. ^{1}H and ^{13}C NMR spectra of the complex **3** in DMSO-d_6

1

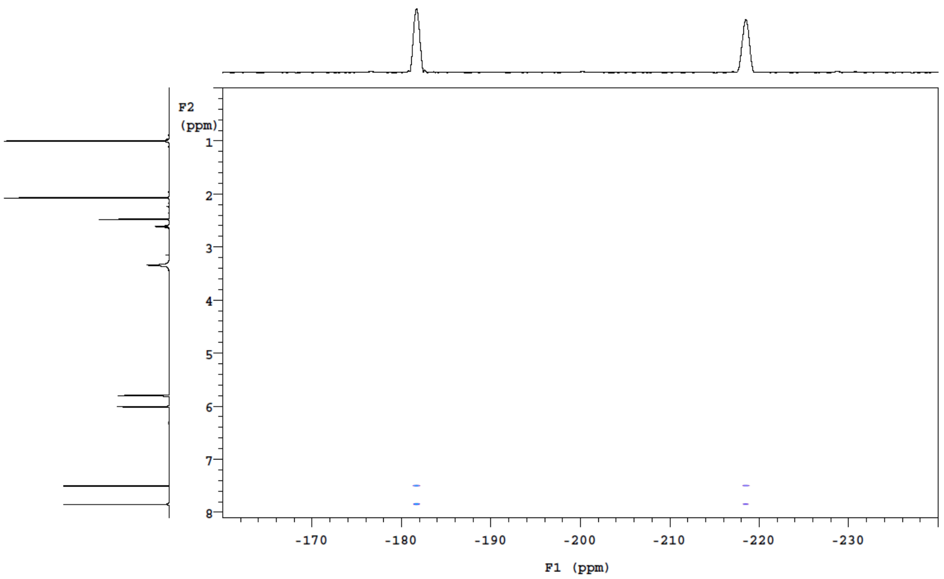

2

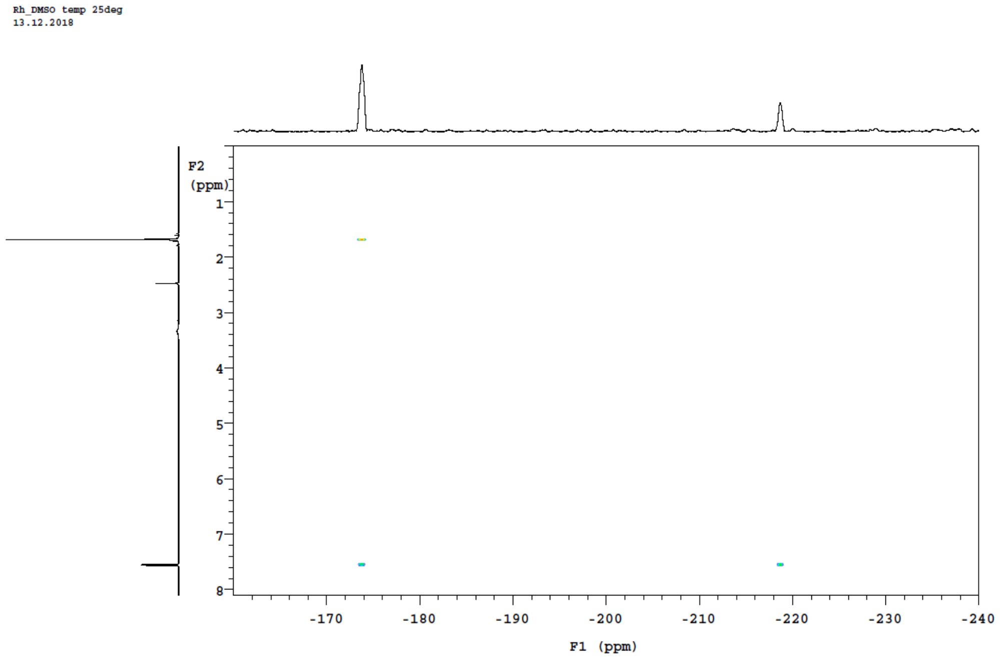

3

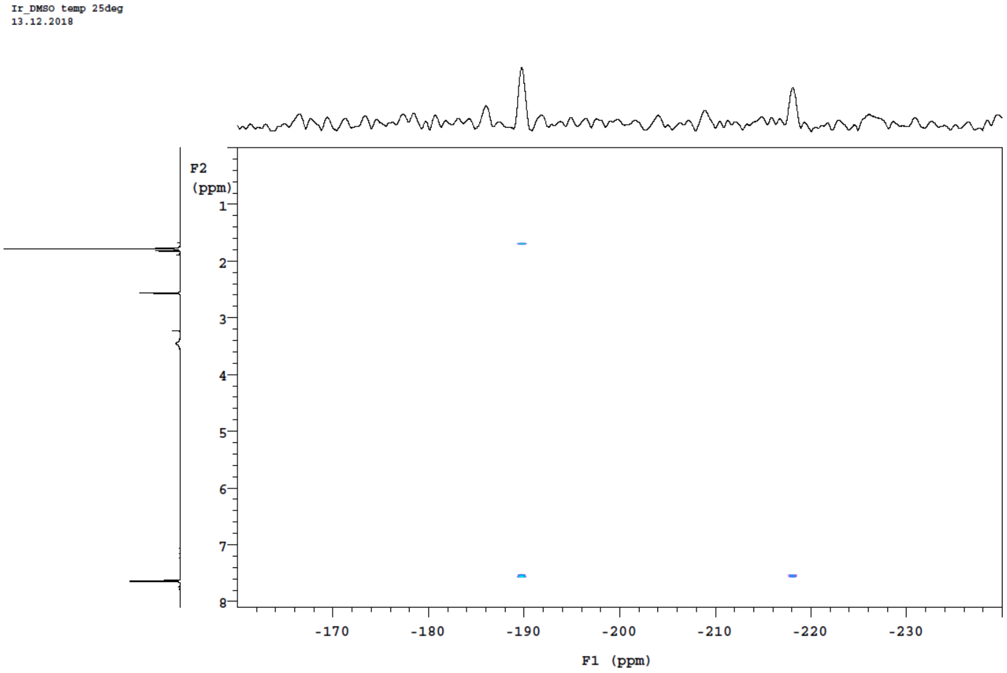

Fig. S8. HMBC ^{15}N NMR spectra of analysed complexes **1-3**.

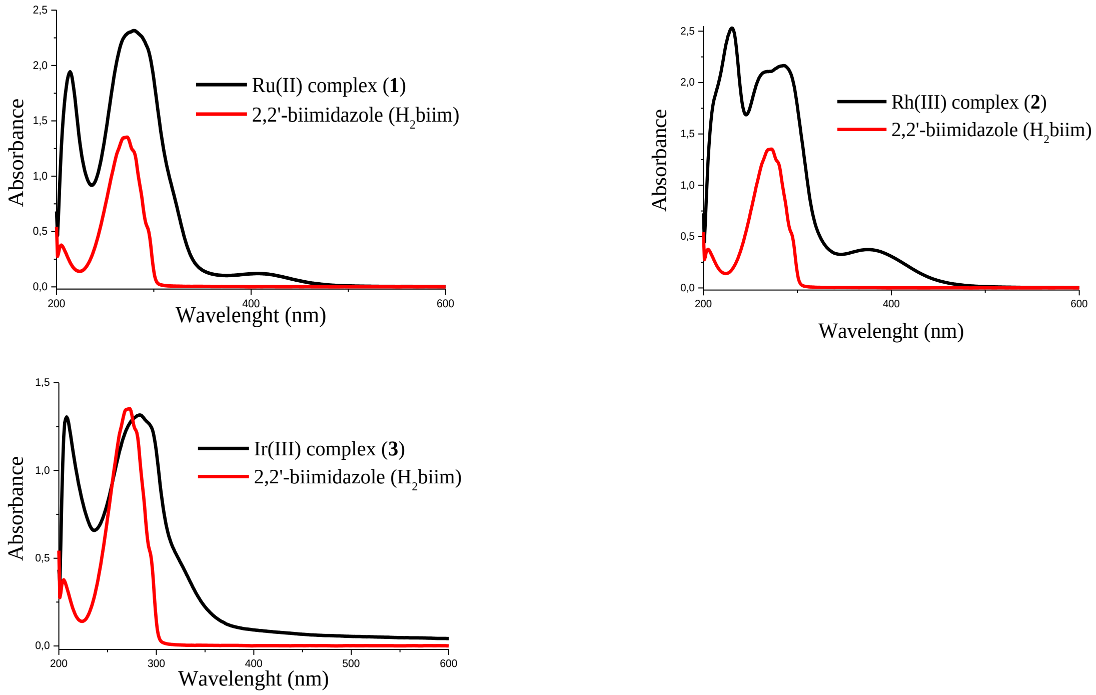

Fig. S9. UV-Vis spectra of the complexes **1-3** in methanol.

Table S3. UV-Vis spectroscopic data for **1-3** complexes and free ligand

	Transition	λ [nm] (ε [dm^3/mol · cm])
H_2biim (ligand)	*π→π*/ n→π**	205 (3772), 272 (13519)
**1**	*π→π*/ n→π**	214 (19436), 280 (23149)
	MLCT dπ(Ru) → π*(L)	410 (1199)
**2**	*π→π*/ n→π**	230 (25301), 285 (21656)
	MLCT dπ(Rh) → π*(L)	377 (3734)
**3**	*π→π*/ n→π**	208 (12998), 283 (13102)
	MLCT dπ(Ir) → π*(L)	330 (4171)

1

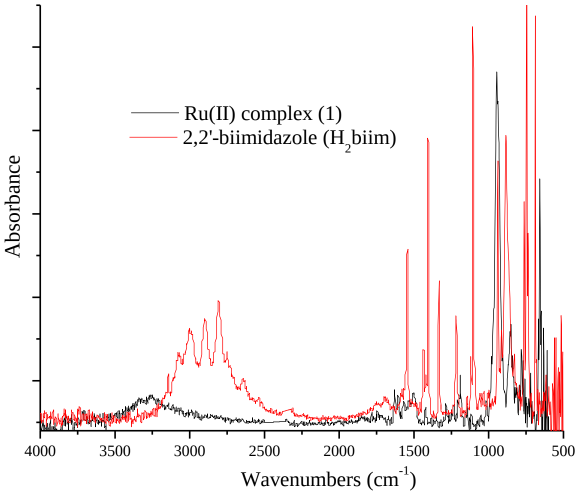

2

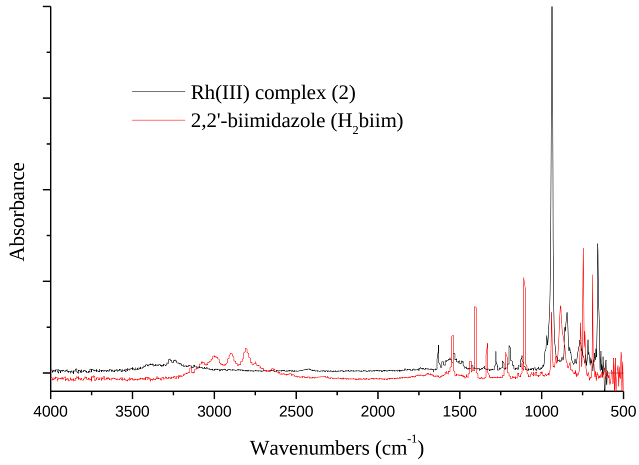

3

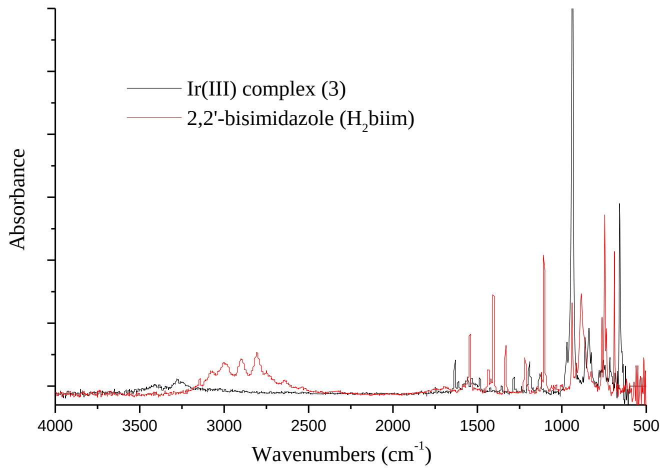

Fig. S10. FTIR spectra of obtained complexes **1-3**.

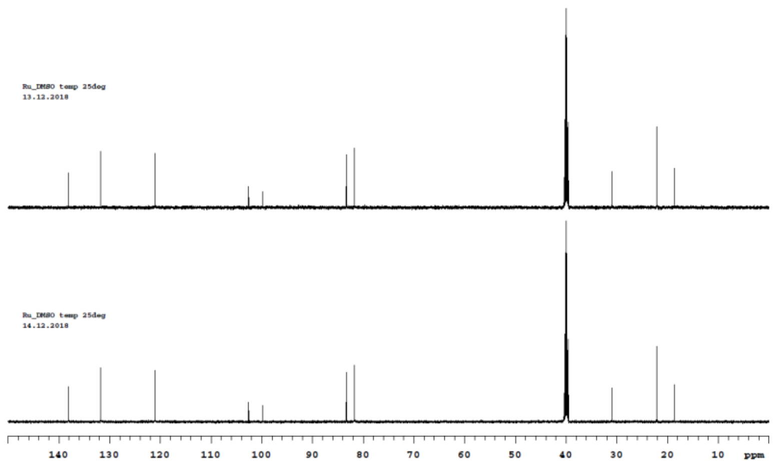

a)

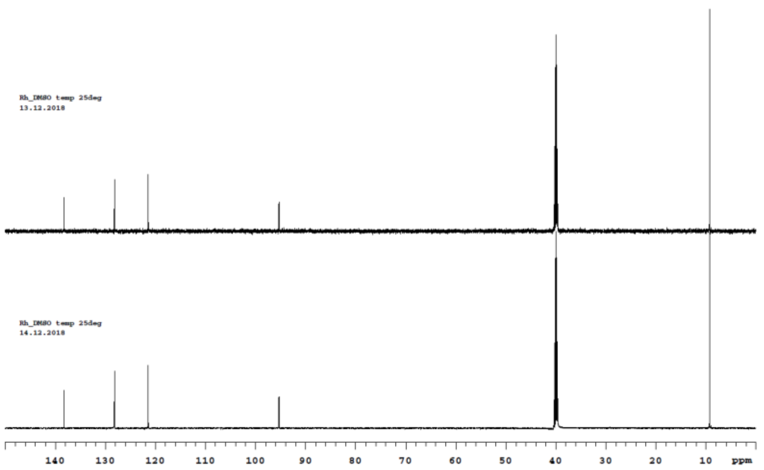

b)

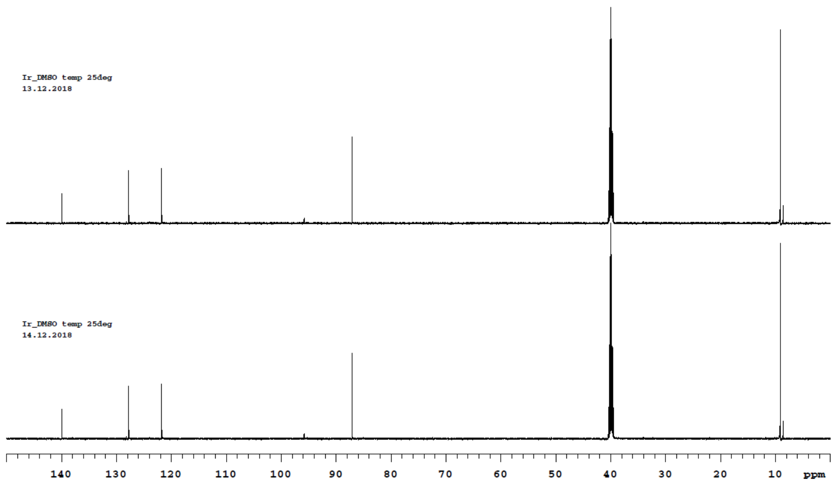

c)

Fig. S11. The time dependent ^{13}C NMR spectra of the complexes (a) **1**, (b) **2**, (c) **3** in DMSO-d_6.

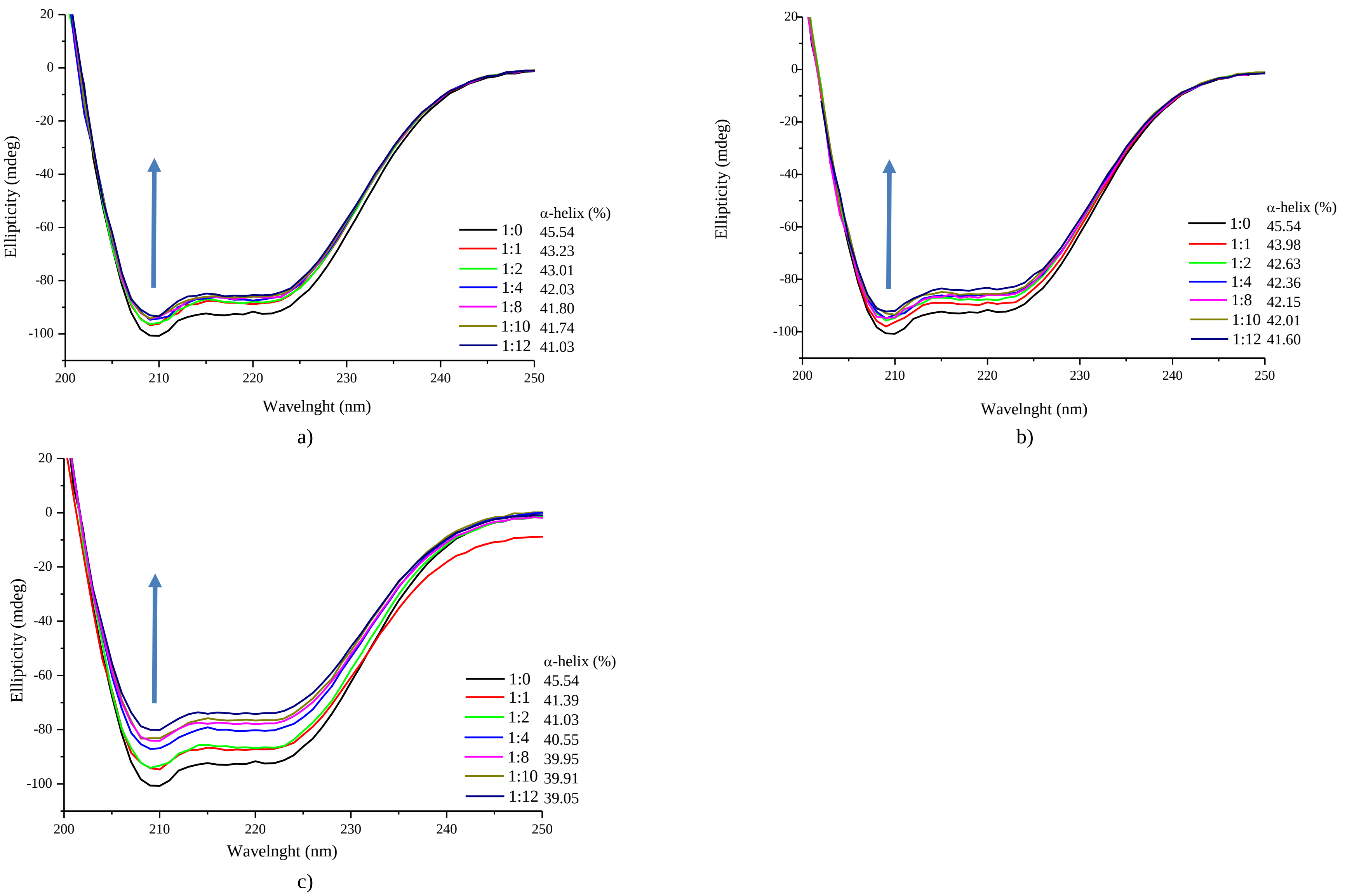

Fig. S12. CD spectra of HSA (10 μM) incubated 24 h with complex **1** (a), **2** (b) and **3** (c) at different [HSA]/[complex] ratios at 37ºC

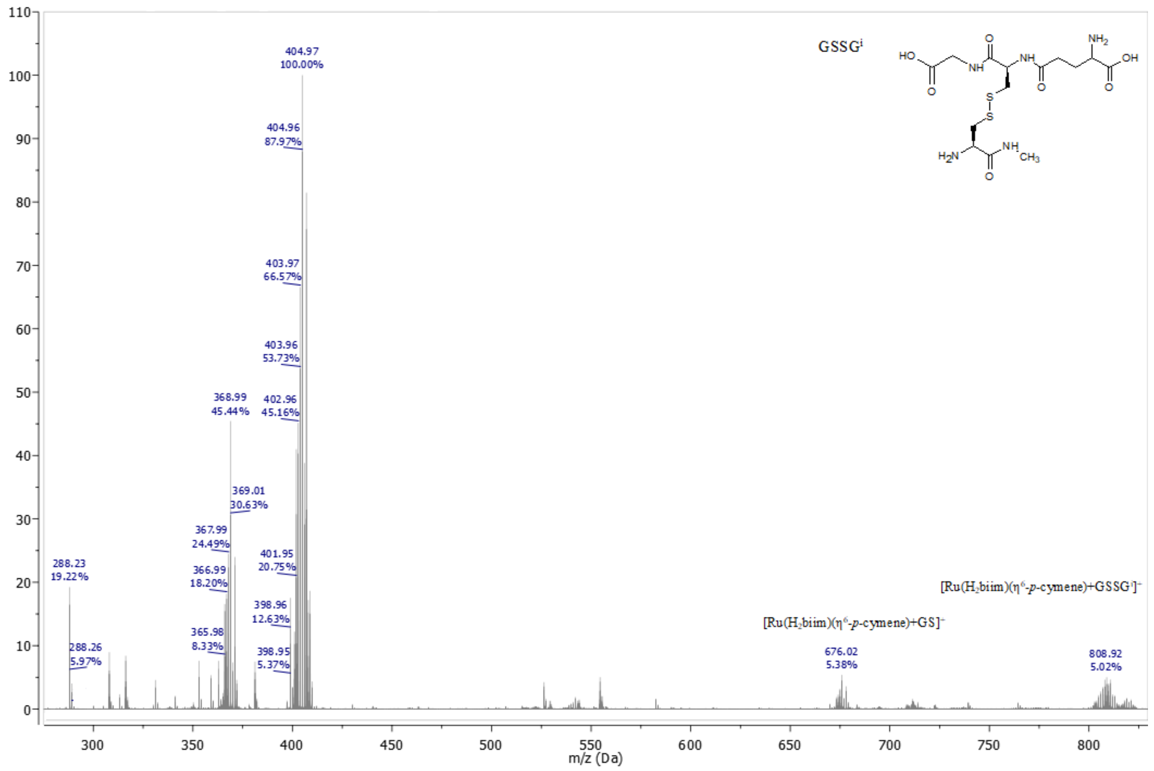
GSSG^i
[Ru(H2biim)(η6-p-cymene)+GSSG^i]−
[Ru(H2biim)(η6-p-cymene)+GS]−
404.97 100.00%
404.96 87.97%
403.97 66.57%
403.96 53.73%
402.96 45.16%
368.99 45.44%
369.01 30.63%
367.99 24.49%
366.99 18.20%
401.95 20.75%
398.96 12.63%
365.98 8.33%
398.95 5.37%
288.23 19.22%
288.26 5.97%
676.02 5.38%
808.92 5.02%
m/z (Da)
a)

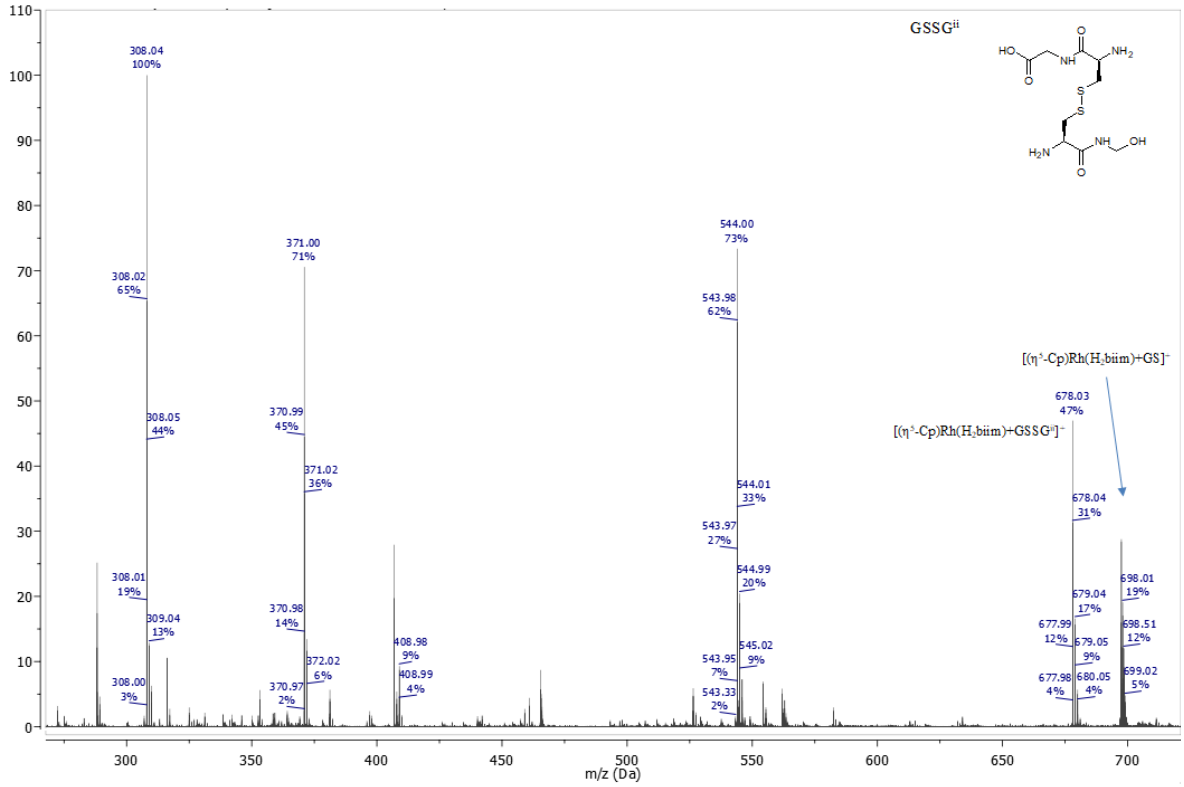
GSSG^ii
[(η5-Cp)Rh(H2biim)+GS]−
[(η5-Cp)Rh(H2biim)+GSSG^ii]−
308.04 100%
308.02 65%
308.05 44%
308.01 19%
309.04 13%
308.00 3%
371.00 71%
370.99 45%
371.02 36%
370.98 14%
372.02 6%
370.97 2%
408.98 9%
408.99 4%
544.00 73%
543.98 62%
544.01 33%
543.97 27%
544.99 20%
545.02 9%
543.95 7%
543.33 2%
678.03 47%
678.04 31%
679.04 17%
677.99 12%
679.05 9%
677.98 4%
680.05 4%
698.01 19%
698.51 12%
699.02 5%
m/z (Da)
b)

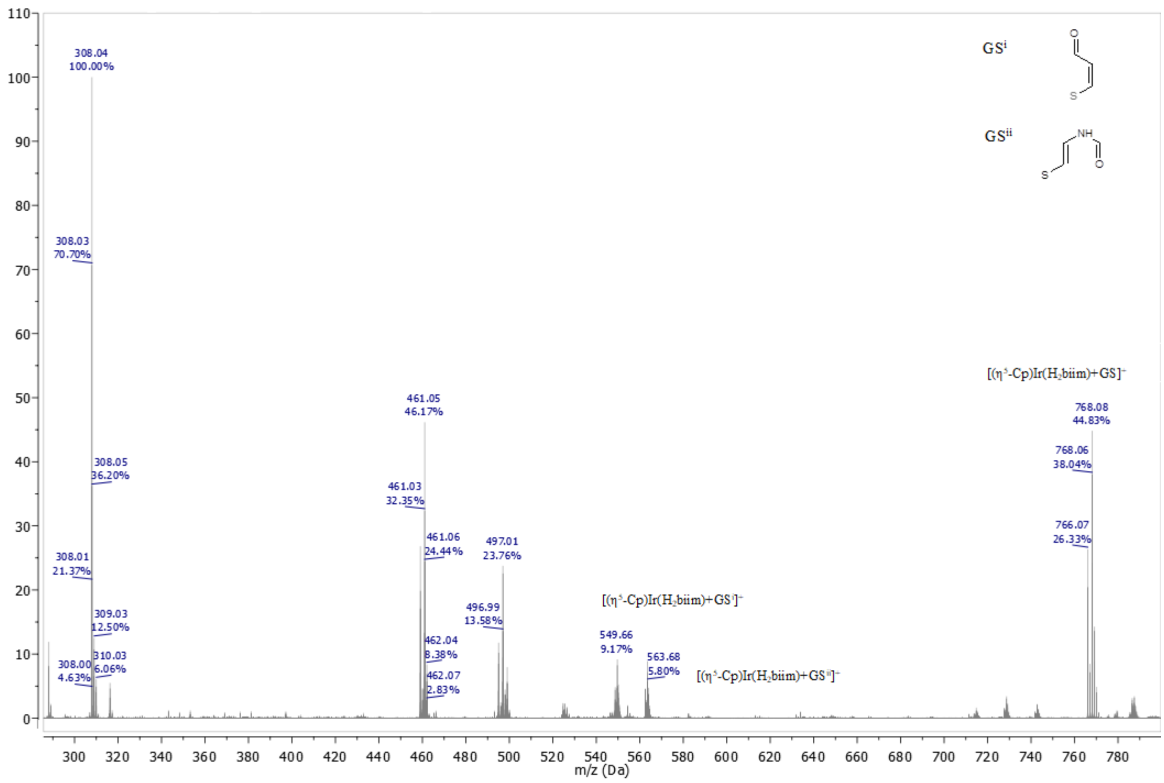
GS^i
GS^ii
[(η⁵-Cp)Ir(H₂biim)+GS]⁻
[(η⁵-Cp)Ir(H₂biim)+GS^i]⁻
[(η⁵-Cp)Ir(H₂biim)+GS^ii]⁻
m/z (Da)

c)

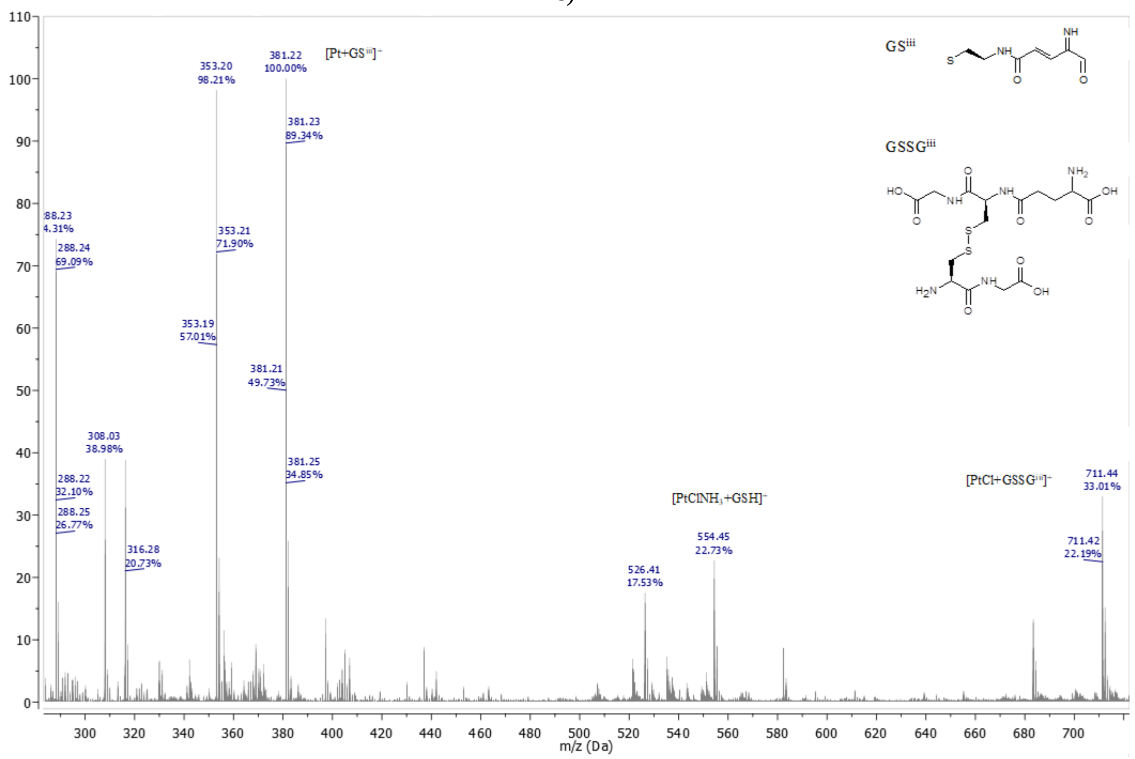
[Pt+GS^iii]⁻
GS^iii
GSSG^iii
[PtClNH₃+GSH]⁻
[PtCl+GSSG^iii]⁻
m/z (Da)

d)

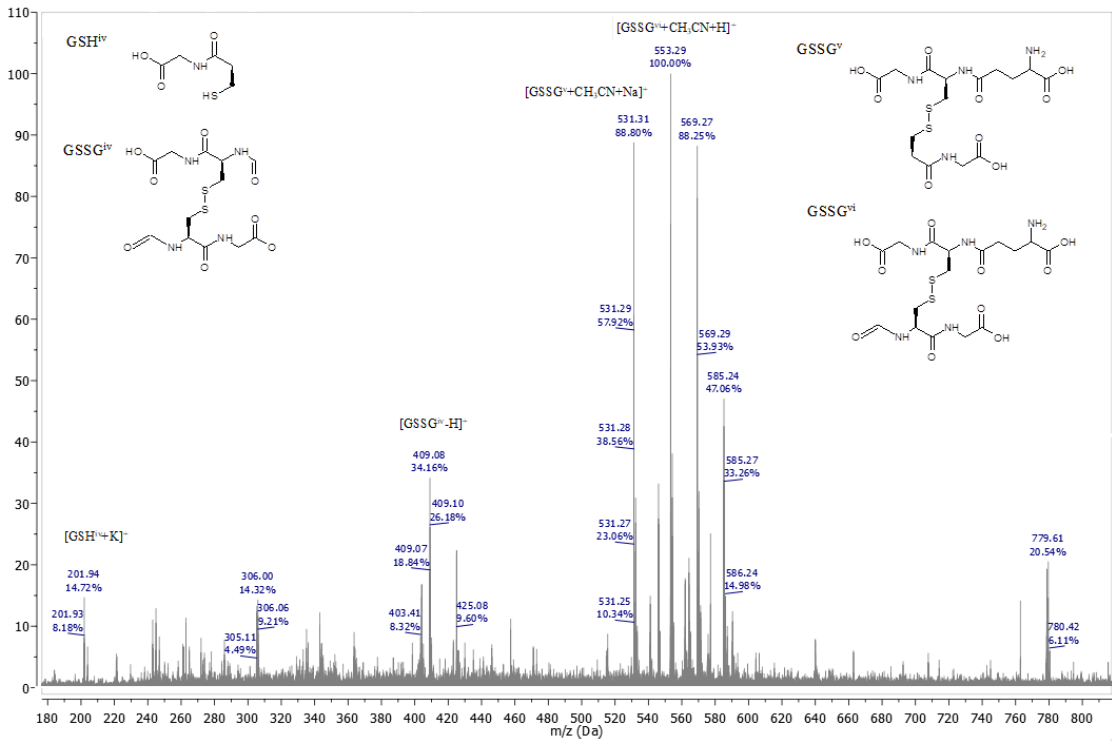

e)

Fig. S13. ESI mass spectra (+ ion mode) obtained after incubation for 24 h at room temperature of the mixture of GSH with appropriate complex a) **1**, b) **2**, c) **3** or d) cisplatin and e) free GSH.

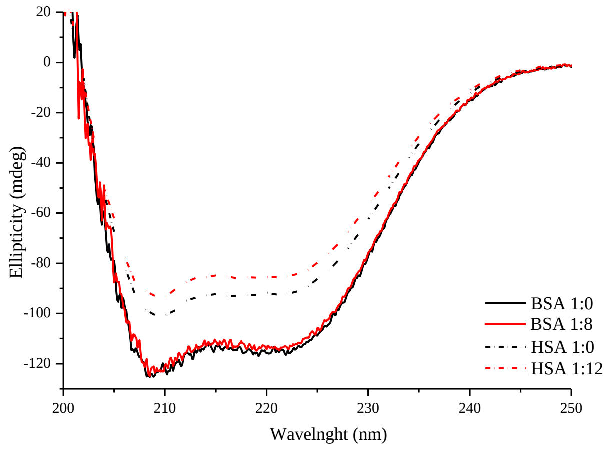

a)

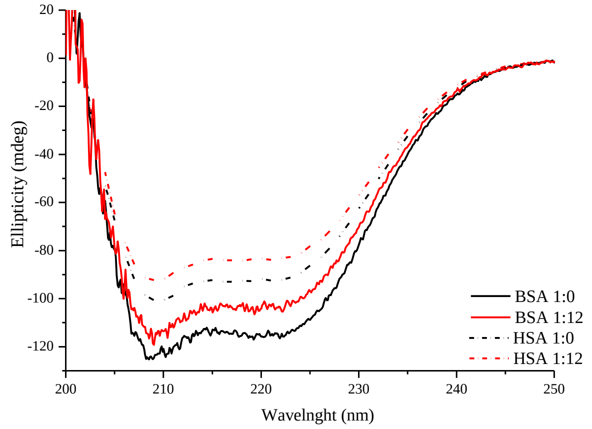

b)

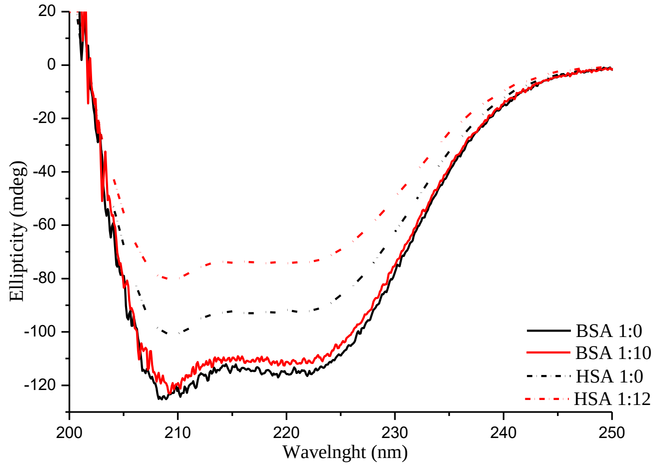

c)

Fig. S14. Overview of the spectra of BSA vel HAS with analysed complexes: a) **1**, b) **2**, c) **3**.
