# Supplementary material for: Similarities and differences in d6 low-spin ruthenium, rhodium and iridium half-sandwich complexes: synthesis, structure, cytotoxicity and interaction with biological targets
Source: J Biol Inorg Chem. 2019 May 21;24(4):591–606. doi: 10.1007/s00775-019-01665-2 (PMC6598960; doi:10.1007/s00775-019-01665-2)

checkCIF/PLATON report

Structure factors have been supplied for datablock(s) I

THIS REPORT IS FOR GUIDANCE ONLY. IF USED AS PART OF A REVIEW PROCEDURE FOR PUBLICATION, IT SHOULD NOT REPLACE THE EXPERTISE OF AN EXPERIENCED CRYSTALLOGRAPHIC REFEREE.

No syntax errors found. CIF dictionary Interpreting this report

Datablock: I

```
Bond precision:  C-C = 0.0158 A          Wavelength=0.71073

Cell:            a=12.0477(8)    b=14.8587(7)      c=22.9202(15)
                 alpha=90        beta=96.246(5)    gamma=90
Temperature:     120 K
```

	Calculated	Reported
Volume	4078.7(4)	4078.7(4)
Space group	P 21/n	P 21/n
Hall group	-P 2yn	-P 2yn
Moiety formula	2(C16 H20 Cl N4 Ru), 2(F6 P), H2 O	2(C16 H20 Cl N4 Ru), 2(F6 P), H2 O
Sum formula	C32 H42 Cl2 F12 N8 O P2 Ru2	C32 H42 Cl2 F12 N8 O P2 Ru2
Mr	1117.72	1117.71
Dx,g cm-3	1.820	1.820
Z	4	4
Mu (mm-1)	1.045	1.045
F000	2232.0	2232.0
F000’	2225.07	
h,k,lmax	14,18,27	14,18,27
Nref	7582	7473
Tmin,Tmax	0.731,0.786	0.771,0.839
Tmin’	0.716	

```
Correction method= # Reported T Limits: Tmin=0.771 Tmax=0.839
AbsCorr = NUMERICAL

Data completeness= 0.986          Theta(max)= 25.499

R(reflections)= 0.0730( 6434)     wR2(reflections)= 0.2300( 7473)

S = 1.143                   Npar= 538
```

The following ALERTS were generated. Each ALERT has the format
test-name_ALERT_alert-type_alert-level.
Click on the hyperlinks for more details of the test.

Alert level B

```
PLAT934_ALERT_3_B Number of (Iobs-Icalc)/SigmaW > 10 Outliers ....          5 Check
```

Alert level C

```
PLAT001_ALERT_1_C No _shelx_res_file DataName Found in SHELXL CIF      Please Do !
PLAT094_ALERT_2_C Ratio of Maximum / Minimum Residual Density ....       2.03 Report
PLAT244_ALERT_4_C Low    'Solvent' Ueq as Compared to Neighbors of        P1 Check
PLAT250_ALERT_2_C Large U3/U1 Ratio for Average U(i,j) Tensor ....        2.5 Note
PLAT250_ALERT_2_C Large U3/U1 Ratio for Average U(i,j) Tensor ....        2.1 Note
PLAT342_ALERT_3_C Low Bond Precision on  C-C Bonds ..............    0.01581 Ang.
PLAT906_ALERT_3_C Large K Value in the Analysis of Variance ......     10.205 Check
PLAT906_ALERT_3_C Large K Value in the Analysis of Variance ......      2.127 Check
PLAT910_ALERT_3_C Missing # of FCF Reflection(s) Below Theta(Min).          7 Note
PLAT911_ALERT_3_C Missing FCF Refl Between Thmin & STh/L=    0.600         98 Report
PLAT918_ALERT_3_C Reflection(s) with I(obs) much Smaller I(calc) .         17 Check
PLAT939_ALERT_3_C Large Value of Not (SHELXL) Weight Optimized S .      13.70 Check
PLAT971_ALERT_2_C Check Calcd Resid. Dens.  1.12A   From F8              2.15 eA-3
PLAT971_ALERT_2_C Check Calcd Resid. Dens.  1.52A   From C35             1.73 eA-3
PLAT971_ALERT_2_C Check Calcd Resid. Dens.  1.02A   From Ru1             1.68 eA-3
PLAT971_ALERT_2_C Check Calcd Resid. Dens.  0.89A   From C35             1.53 eA-3
PLAT977_ALERT_2_C Check Negative Difference Density on H15              -0.38 eA-3
PLAT977_ALERT_2_C Check Negative Difference Density on H30C             -0.42 eA-3
PLAT977_ALERT_2_C Check Negative Difference Density on H38C             -0.41 eA-3
PLAT978_ALERT_2_C Number C-C Bonds with Positive Residual Density.          0 Info
```

Alert level G

```
PLAT007_ALERT_5_G Number of Unrefined Donor-H Atoms ..............          6 Report
PLAT083_ALERT_2_G SHELXL Second Parameter in WGHT  Unusually Large     135.61 Why ?
PLAT244_ALERT_4_G Low    'Solvent' Ueq as Compared to Neighbors of        P2 Check
PLAT333_ALERT_2_G Large Aver C6-Ring C-C Dist C21      -C26      .       1.42 Ang.
PLAT432_ALERT_2_G Short Inter X...Y Contact  F1       ..C23              2.94 Ang.
                                         1/2-x,-1/2+y,1/2-z  =      2_545 Check
PLAT912_ALERT_4_G Missing # of FCF Reflections Above STh/L=  0.600          4 Note
PLAT933_ALERT_2_G Number of OMIT Records in Embedded .res File ...          2 Note
```

---

 0 **ALERT level A** = Most likely a serious problem - resolve or explain
 1 **ALERT level B** = A potentially serious problem, consider carefully
20 **ALERT level C** = Check. Ensure it is not caused by an omission or oversight
 7 **ALERT level G** = General information/check it is not something unexpected

```
 1 ALERT type 1 CIF construction/syntax error, inconsistent or missing data
15 ALERT type 2 Indicator that the structure model may be wrong or deficient
 8 ALERT type 3 Indicator that the structure quality may be low
 3 ALERT type 4 Improvement, methodology, query or suggestion
 1 ALERT type 5 Informative message, check
```

---

checkCIF publication errors

Alert level A

```
PUBL006_ALERT_1_A  _publ_requested_journal is missing
            e.g. 'Acta Crystallographica Section C'
PUBL012_ALERT_1_A  _publ_section_abstract is missing.
            Abstract of paper in English.
```

Alert level G

```
PUBL017_ALERT_1_G The _publ_section_references section is missing or
            empty.
```

```
   2 ALERT level A = Data missing that is essential or data in wrong format
   1 ALERT level G = General alerts. Data that may be required is missing
```

Publication of your CIF

You should attempt to resolve as many as possible of the alerts in all categories. Often the minor alerts point to easily fixed oversights, errors and omissions in your CIF or refinement strategy, so attention to these fine details can be worthwhile. In order to resolve some of the more serious problems it may be necessary to carry out additional measurements or structure refinements. However, the nature of your study may justify the reported deviations from journal submission requirements and the more serious of these should be commented upon in the discussion or experimental section of a paper or in the "special_details" fields of the CIF. *checkCIF* was carefully designed to identify outliers and unusual parameters, but every test has its limitations and alerts that are not important in a particular case may appear. Conversely, the absence of alerts does not guarantee there are no aspects of the results needing attention. It is up to the individual to critically assess their own results and, if necessary, seek expert advice.

If level A alerts remain, which you believe to be justified deviations, and you intend to submit this CIF for publication in a journal, you should additionally insert an explanation in your CIF using the Validation Reply Form (VRF) below. This will allow your explanation to be considered as part of the review process.

Validation response form

Please find below a validation response form (VRF) that can be filled in and pasted into your CIF.

```
# start Validation Reply Form
_vrf_PUBL006_GLOBAL
;
PROBLEM: _publ_requested_journal is missing
RESPONSE: ...
;
_vrf_PUBL012_GLOBAL
;
PROBLEM: _publ_section_abstract is missing.
RESPONSE: ...
;
# end Validation Reply Form
```

If you wish to submit your CIF for publication in Acta Crystallographica Section C or E, you should upload your CIF via the web. If you wish to submit your CIF for publication in IUCrData you should upload your CIF via the web. If your CIF is to form part of a submission to another IUCr journal, you will be asked, either during electronic submission or by the Co-editor handling your paper, to upload your CIF via our web site.

PLATON version of 19/10/2018; check.def file version of 15/10/2018

Datablock I - ellipsoid plot

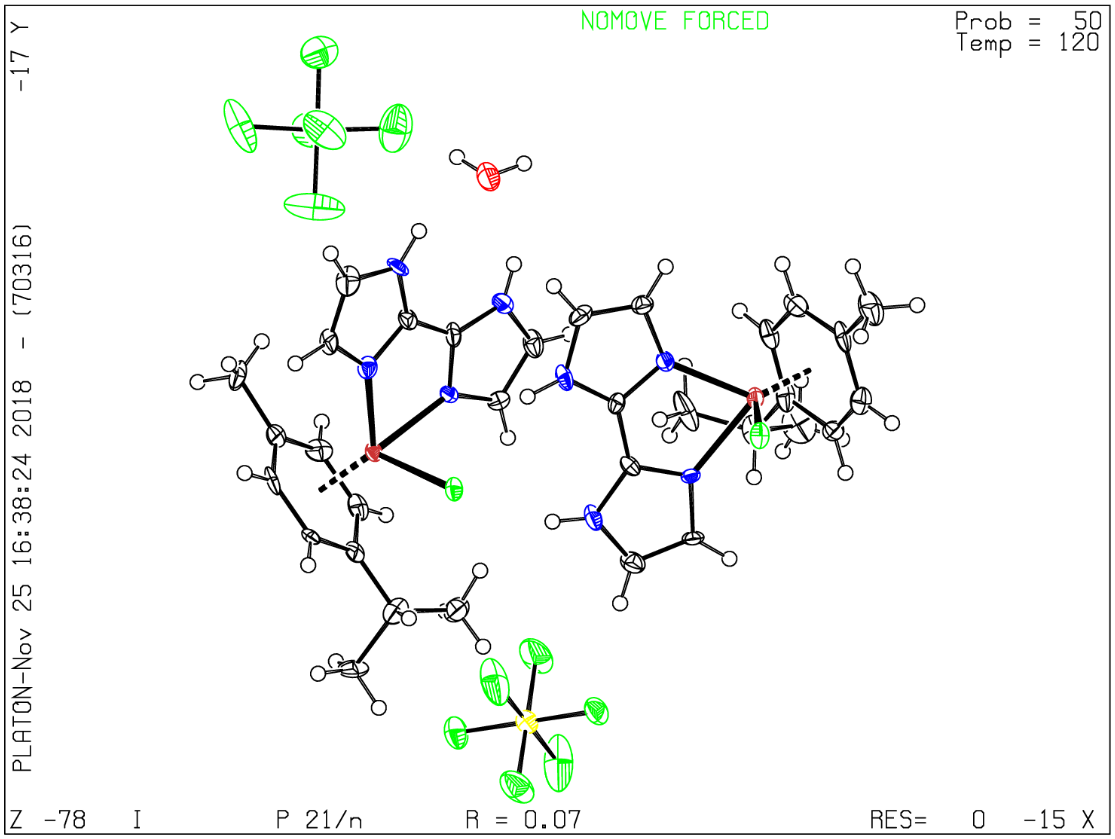

Supplement: Supplementary file 2 — Supplementary material 2 (PDF 143 kb) [file 775_2019_1665_MOESM2_ESM.pdf]
